# Numerical simulation of heat transfer and fluid characteristics in micro-channels with obliquely arranged capsule-shaped ribs and optimization study of structural parameters

**DOI:** 10.1371/journal.pone.0346804

**Published:** 2026-04-17

**Authors:** Junkai Yao, Chongyuan Li, Yuhao Shen, Yin Wei, Hao Zheng, Haichao Feng, Gaowei Shao, Jiexiang Yang, Yuefeng Li

**Affiliations:** 1 School of Science, Shanghai Institute of Technology, Shanghai, China; 2 Kaikan Technology (Changzhou) Co., Ltd, Jiangsu, China; 3 Shaoxing Shangrui Optoelectronics Technology Co., Ltd, Zhejiang, China; GH Raisoni College of Engineering and Management Pune, INDIA

## Abstract

To enhance the cooling performance of high-power laser diodes, this paper proposes a novel micro-channel design that incorporates oblique capsule-shaped ribs. By integrating computational fluid dynamics(CFD) simulations with a non-dominated sorting genetic algorithm II optimized through Gaussian process regression (GPR) and deep neural networks (DNN), this study investigates the influence of fin geometric parameters (*L*_*r*_, *W*_*r*_, *H*_*r*_, *L*_*s*_, and fin shape) on thermal-hydraulic performance under turbulent conditions. The results demonstrate that the ribs perturb the boundary layer and generate vortices, significantly enhancing heat transfer, as evidenced by an increase in the Nusselt number (*Nu*). At a Reynolds number (*Re*) of 11353, the optimized capsule-shaped rib configuration with dimensions (*L*_*r*_ = 2.4928 mm, *W*_*r*_ = 1.3944 mm, *H*_*r*_ = 1.3490 mm, *L*_*s*_ = 2.9742 mm) delivers optimal performance, compared to a smooth channel, it reduces the temperature standard deviation (*σ*) by 3.1%, decreases thermal resistance (*R*_*TH*_) by 8.3%, and increases *Nu* by 20.2%. Although the pressure drop (*f*) increases by 15.3%, the *PEC* improves by 15.6%. This study provides a theoretical foundation and engineering strategy for the optimized design of heat sinks in high-power electronic devices.

## 1. Introduction

Recently, the rapid advancement of contemporary electronic technology has resulted in a continual increase in the power heat flux density of electronic devices, which are evolving towards higher integration and miniaturization. An increase in operating temperature by 10°C leads to a doubling of the failure rate of electronic components. The heat dissipation requirements for traditional integrated circuits have reached 100 W/cm², while in very large-scale integrated circuits, they have surpassed 1000 W/cm^2^ [1,2]. High-power laser diodes are critical components of semiconductor lasers and generate significant heat during operation [3]. To ensure sufficient heat dissipation and enhance their longevity, there is an urgent need for effective cooling solutions [4–7].

The technology of cooling through micro-channels was initially introduced by Tuckerman and Pease [8], offering advantages such as increased heat transfer efficiency, a compact design, and a large specific surface area. These attributes make this technology an effective solution for managing heat dissipation in high-temperature electronic devices [9]. Traditional passive techniques employed in micro-channels include the addition of substances to the fluid [10], modifications to the internal channel architecture [11,12], alterations to the channel surfaces [13], and the incorporation of damping components such as fins, ribs, cavities and various inserts [14–20]. A study conducted by Peng and Peterson [21] investigated heat transfer in single-phase forced convection and the corresponding flow properties, highlighting that geometric configurations significantly influence these thermal and flow characteristics. This finding underscores the necessity for enhanced surface contact area or improved surface convective heat transfer coefficients to increase the heat transfer coefficient (HTC).

Various designs for micro-channel heat sinks distinguish them from traditional micro-channels. Examples include rectangular flow-blocking structures [22–24], corrugated designs [25,26], V-tapered-baffles [27], flat groove formations [28–31], convex or slanted geometries [32,33], and complex baffle geometries meant to optimize flow topology [34,35]. Enhancing the heat transfer coefficient (HTC) can also be achieved by modifying the surface characteristics at the fluid-channel interface, utilizing features such as slot-like topography and indentations [36,37]. Furthermore, the arrangement of these turbulators, such as staggered versus in-line configurations, plays a critical role in thermal-hydraulic performance [38]. Kishimoto et al. [39] introduced an interleaved fin configuration that reduced temperature fluctuations at nodes by 25% compared to standard micro-channels. Jasperson et al. [40] evaluated the thermal efficiency and pressure loss between traditional channels and staggered micro-needle heat sinks, revealing that, at a liquid flow rate of 60 g/min, the staggered micro-needle design exhibited lower convective thermal resistance while incurring a higher pressure drop. Lee et al. [41] proposed a slanted fin design that mitigates boundary layer growth, improving the average heat transfer coefficient by up to 80%. Soodphakdee et al. [42] examined the thermal conductivity of fins with varying cross-sectional shapes (cylindrical, square, and elliptical) across different arrangements. Their findings suggest that staggered elliptical fins provide optimal thermal conductivity under low-pressure drop conditions, whereas staggered cylindrical fins excel under high-pressure drop scenarios. Colgan et al. [43] investigated staggered fins positioned on a silicon substrate, reporting that the heat dissipation capacity reached 500 W/cm², with thermal conductivity measuring 500,000 W/m^2^·K, marking the highest value documented [44,45]. In conclusion, the staggered fin ribs design demonstrates superior thermal conductivity compared to other micro-channel configurations at the same pressure drop level.

The current designs of liquid-cooled heat sink primarily consist of fin-type and pin-type configurations. Chyu [46] investigated the heat dissipation capabilities of pin arrays with varying heights and height-to-diameter ratios, revealing that the average heat transfer rate enhances as either the pin height or height-to-diameter ratio increases. Wang [47] employed a micro-channel heat sink setup that incorporates micro-scale fins and slots, performing both experimental and numerical analyses to evaluate the cooling efficiency of fin-slot micro-channels in comparison to standard smooth rectangular micro-channels. Shwaish [48] examined the thermal performance of serrated fin radiators in operation, analyzing various *Re* while assessing temperature distributions, heat transfer coefficients, *Nu*, pressure drops, and maximum temperatures within the radiator’s fins. Jajja [49] investigated the impact of five distinct fin spacings alongside a flat plate fin on the thermal management of high-power microprocessors. Muhammad [50] extended this research by examining the thermal-hydraulic performance of micro-channel heat sinks featuring diverse fin designs. Kumar [51] studied the flow and heat transfer behavior in a micro-channel heat sink with an innovative inlet/outlet configuration under varying inlet flow angles. Xia [52] explored three different inlet/outlet configurations and channel cross-sectional shapes to enhance the heat transfer efficiency of micro-channel heat sinks. Ma [53] optimized the design of micro-channel heat sinks tailored to specific chip geometries by adjusting the ratios of chip width to channel spacing, channel width to spacing, and aspect ratios. Tan [54] designed four topological structures to investigate the influence of micro-channel topology on chip cooling heat transfer performance, conducting experiments on straight and spider-web-shaped micro-channels utilizing 3D printing technology.

Turbulence within micro-channels is primarily classified into two categories: those occurring at the base and those located along the channel walls. Typically, the ribs positioned at the base are situated near the central region, enhancing heat transfer while generating significant flow resistance [55]. Conversely, ribs placed on the wall surfaces may not achieve the same level of heat transfer efficiency, although they can enhance hydraulic performance. A numerical investigation by Di Capua H et al. [56] on heat transfer and flow within micro-channels featuring forward-facing triangular ribs on the sidewalls demonstrated a concurrent increase in both heat transfer and flow resistance. Feng et al. [57] reported on a microchannel that features staggered triangular ribs arranged on both the bottom and top surfaces. Numerical results demonstrated that this configuration can generate vortices, enhance heat transfer, and maintain reasonable flow resistance. At a Reynolds number of 639, the maximum performance evaluation criterion (*PEC*) reached 1.502. However, regions of extremely low flow velocity exist behind the right-angled fins, which partially compromise heat transfer efficiency. Al-Asadi et al. [58] compared the heat transfer performance of microchannels with quarter-cylindrical and semi-cylindrical fins on the bottom surface. The study found that, in most cases, quarter-cylindrical fins did not effectively reduce thermal resistance, primarily due to extensive low-velocity flow zones behind them. In contrast, microchannels with semi-cylindrical fins exhibited significantly enhanced heat transfer performance. This indicates that fins with an expanding-contracting cross-section may offer superior microchannel performance compared to those with a purely expanding cross-section, as they reduce low-velocity flow zones. This conclusion aligns with the findings of Lory and Wafai [59]. Furthermore, Li et al. [60] designed MCHS integrated with grooved sidewall fins, with their experimental findings demonstrating that symmetric vortices promote turbulence, thereby enhancing the thermal performance of the MCHS.

In summary, this paper proposes a microchannel design featuring diagonally staggered capsule-shaped fins. This design aims to achieve efficient heat transfer by inducing longitudinal vortices while minimizing flow resistance. These fins are engineered with an expanding and contracting cross-sectional shape to reduce low-velocity flow regions. Numerical simulations are conducted to analyze the flow field characteristics within the finned microchannel. Additionally, the effects of fin width, height, length, and spacing on the microchannel’s thermal and hydraulic performance are thoroughly investigated. Data analysis and mining are performed using deep artificial neural network algorithms in conjunction with the NSGA-II genetic algorithm. Finally, the TOPSIS method is employed to identify an optimal compromise solution from the Pareto front, balancing thermal and hydraulic performance. This research provides both theoretical and data-driven support for thermal management in power modules for consumer electronics, artificial intelligence computing, and new energy applications.

## 2. Numerical simulation

### 2.1. Research process

**Fig 1:**
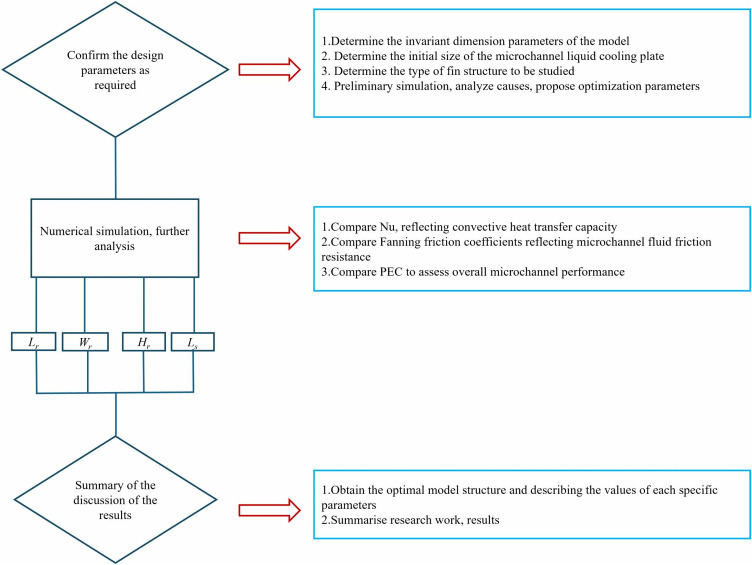
Flow chart of the study.

(Fig 1) illustrates the research workflow of this study. Based on the operational and thermal management requirements of the laser chip, the fixed dimensions of the model were established. The initial dimensions of the micro-channel liquid cooling plate were defined, and its structural configuration was selected. Subsequently, a three-dimensional model of the designed cooling plate was constructed. The model’s boundary conditions were then input into CFD software for numerical simulation. Under turbulent flow conditions, the effects of Lr, Wr, Hr, and Re, Nu, f, and PEC were analyzed to identify the optimal structural design. Finally, neural networks and genetic algorithms were employed to determine the optimal structural parameter combinations.

### 2.2. Physical model and boundary conditions

#### 2.2.1. Physical model.

**Fig 2:**
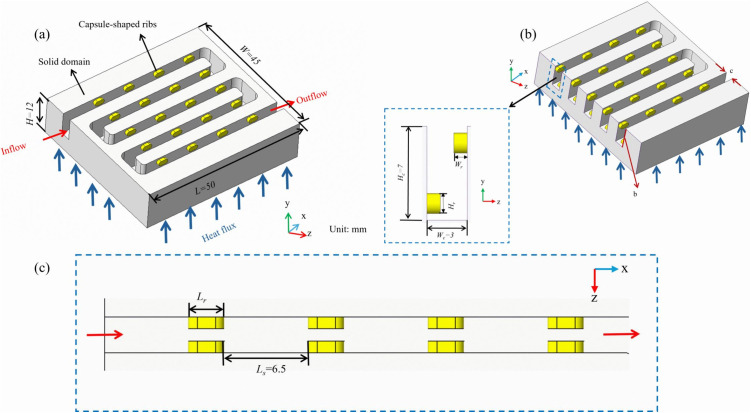
Schematic diagrams and dimensions of the designed micro-channel. a Physical model, b Cross-sectional view, c Top view.

Micro-channels featuring capsule-shaped ribs are illustrated in (Fig 2a), where two rows of these ribs are arranged diagonally across the cross-section. The computational model is divided into solid and fluid domain, where the solid domain comprises the capsule-shaped rib structures. As shown in (Fig 2), The dimensions of the solid computational domain are length *L* = 50 mm, width *W* = 45 mm, and height *H* = 12 mm. In contrast, the fluid computational domain has a height *H* = 7 mm and a width *W* = 3 mm. The rib spacing is defined as *L*_*s*_ = 6.5 mm. The rib design parameters investigated are *L*_*r*_, *W*_*r*_, and *H*_*r*_, with values listed in Table 1.

**Table 1:** Investigated parameter values of the ribs.

Parameter	Value(mm)
*L*_*r*_	0.5 1 1.5 2 2.5
*W*_*r*_	0.6 0.8 1 1.2 1.4
*H*_*r*_	1.5 2 2.5 3 3.5

#### 2.2.2. Boundary conditions.

The material properties and boundary conditions for this study are shown in Tables 2 and Table 3:

**Table 2:** Material Properties.

Item	Material	Value	Unit
Base material	Cu	–	–
Coolant	Water	–	–
Cover material	Cu	–	–
TIM	Glue-13	–	–

**Table 3:** Parameter values of the boundary conditions.

Item	Value	Unit
Pout	101325	Pa
TIM	0.2	mm
Tin	341	K
Vin	1.14	m/s
λ_TIM_ ρ_TIM_	13 1120	W/(m*K) Kg/m^3^

### 2.3. Mathematical model

In this study, several justified assumptions were established prior to the simulation to facilitate accurate numerical computations of heat transfer and fluid flow phenomena:

(1) The fluid flow is single-phase;
(2) Gravity, viscous dissipation, and thermal radiation are neglected;
(3) The flow is three-dimensional, steady, and incompressible;
(4) Based on the operating conditions and rib-induced flow disturbance, the fluid flow in the micro-channel is assumed to be fully turbulent;
(5) The solid material is copper, with a thermal conductivity of approximately 400 W/(m·K). The cooling fluid is deionized water, whose density (*ρ*_*f*_), specific heat (*C*_*p*_), thermal conductivity (k), and dynamic viscosity coefficient (*μ*_*f*_) are temperature-dependent functions, defined as follows:

$$\rho_{f}=\frac{\begin{array}{c} (998.84+18.225T-7.92\times 10^{-3}T^{\text{2}}-5.545\times 10^{-5}T^{\text{3}}\\ +1.498\times 10^{-7}T^{\text{4}}-3.933\times 10^{-10}T^{\text{5}}) \end{array}}{1+1.816\times 10^{-2}T}$$ (1)

$$C_{p}=8958.9-40.535T+0.11243T^{\text{2}}-1.014\times 10^{-4}T^{\text{3}}$$ (2)

$$k_{f}=-0.58166+6.3556\times 10^{-3}T-7.964\times 10^{-6}T^{\text{2}}$$ (3)

$$\mu_{f}=2.414\times 10^{-5}\times 10^{\frac{247.8}{T-140}}$$ (4)

In Equation (1), the unit of *T* is °C. In equations (2), (3), and (4), the unit of *T* is K. The applied boundary conditions are as follows:

(1) The inlet velocity *V*_*in*_ of the micro-channel is 1.14 m/s, and the inlet temperature *T*_*in*_ is 341 K.
(2) Since the outlet of the micro-channel is in contact with the atmosphere, a constant relative pressure *P*_*out*_ = 101.325 kPa is selected.
(3) On the heat sink surface at *z* = 0, the bottom boundary is heated in the form of uniform heat flow:

$$-k_{s}\frac{\alpha T_{s}}{\alpha_{z}}=q=31.27W/cm^{\text{2}}$$ (5)

CFD is an engineering discipline that integrates fluid mechanics and numerical methods to simulate fluid movement by solving conservation equations related to mass, momentum, and energy within a continuous medium. These fundamental flow equations are typically represented by partial differential equations, and the principles governing fluid motion are based on three essential conservation laws: mass, momentum, and energy.

(1) Conservation of Mass:


$$\frac{\partial \rho }{\partial t}+\frac{\partial \left(\rho u\right)}{\partial x}+\frac{\partial \left(\rho \upsilon \right)}{\partial y}+\frac{\partial \left(\rho w\right)}{\partial z}=0$$ (6)

In the formula: *ρ* refers to density; *t* refers to time; *u*, *υ*, *w*, *x*, *y*, *z* refers to velocity vectors in the respective directions.

(2) Conservation of Momentum:


$$\frac{\partial \left(\rho u\right)}{\partial t}+di\upsilon \left(\rho uu\right)=-\frac{\partial p}{\partial x}+\frac{\partial \tau_{xx}}{\partial x}+\frac{\partial \tau_{yx}}{\partial y}+\frac{\partial \tau_{zx}}{\partial z}+F_{x}$$ (7)

$$\frac{\partial \left(\rho \upsilon \right)}{\partial t}+di\upsilon \left(\rho \upsilon u\right)=-\frac{\partial p}{\partial y}+\frac{\partial \tau_{xy}}{\partial x}+\frac{\partial \tau_{yy}}{\partial y}+\frac{\partial \tau_{zy}}{\partial z}+F_{y}$$ (8)

$$\frac{\partial \left(\rho w\right)}{\partial t}+di\upsilon \left(\rho wu\right)=-\frac{\partial p}{\partial z}+\frac{\partial \tau_{xz}}{\partial x}+\frac{\partial \tau_{yz}}{\partial y}+\frac{\partial \tau_{zz}}{\partial z}+F_{y}$$ (9)

In the formula: *P* refers to the pressure on the fluid microelement. *τ*_*xx*_、*τ*_*xy*_、*τ*_*yx*_、*τ*_*yy*_、*τ*_*xz*_、*τ*_*zx*_、*τ*_*zy*_、*τ*_*yz*_、*τ*_*zz*_: The viscous stress component acting on the surface of the fluid element due to molecular viscosity; *F*_*x*_、*F*_*y*_、*F*_*z*_: Body force acting on the fluid element(N).

(3) Conservation of Energy:


$$\begin{array}{c} \frac{\partial \left(\rho T\right)}{\partial t}+\frac{\partial \left(\rho uT\right)}{\partial x}+\frac{\partial \left(\rho \upsilon t\right)}{\partial y}+\frac{\partial \left(\rho wT\right)}{\partial z}\mathrm{\backslash vspace}1mm\\ =\frac{\partial }{\partial x}\left[\frac{k}{c_{p}}\frac{\partial T}{\partial x}\right]+\frac{\partial }{\partial y}\left[\frac{k}{c_{p}}\frac{\partial T}{\partial y}\right]+\frac{\partial }{\partial_{z}}\left[\frac{k}{c_{p}}\frac{\partial T}{\partial z}\right]+S_{T} \end{array}$$ (10)

In this formula, *C*_*p*_ denotes the specific heat capacity at constant pressure, while *T* represents temperature. The variable *k* signifies the thermal conductivity of the fluid. Additionally, *ST* represents the volumetric heat source within the fluid and the viscous dissipation term.

The influence of thermal radiation on temperature distribution is neglected in this simulation. This oversight is primarily due to the system’s structure and the environmental conditions surrounding the liquid cooling plate, such as surface properties, channel configuration, and insulation from the external environment—which significantly limit the effective transmission of thermal radiation. Consequently, convection and conduction dominate the heat transfer. Furthermore, the turbulent and dynamic coolant flow emphasizes the dominance of convection. Under these conditions, radiative heat transfer is negligible compared to convection. Additionally, incorporating radiation would significantly increase model complexity, requiring 3D view factor calculations and emissivity specification. Therefore, neglecting radiation simplifies the model while maintaining sufficient accuracy. This approach ensures the model’s applicability and reliability across a broad spectrum of engineering applications.

#### 2.3.1. Heat conduction.

The chip, comprising various materials, is modeled as a solid entity. Heat transfer within the chip occurs via conduction, governed by Fourier law. The formula for this calculation is presented below:


$$Q_{\text{1}}=-k\cdot A_{\text{1}}\cdot \frac{\sigma t}{\sigma x}$$ (11)

In the formula:

*Q*_*1*_: Total heat transferred between media(w);

*k*: The thermal conductivity coefficient of the calculated heat transfer surface(W/(m·K));

*A*_*1*_: Cross-sectional area perpendicular to the direction of heat transfer(m^2^);

*σt/σx*: Temperature gradient(K/m)；

#### 2.3.2. Thermal convection.

Convective heat transfer describes the heat exchange mechanism between a fluid and a solid surface in direct contact. The equation representing convective heat transfer can be formulated based on Newton’s cooling principle, as illustrated below:


$$Q_{\text{2}}=h\cdot A\cdot \left(t_{w}-t_{f}\right)$$ (12)

In the formula:

*Q*_*2*_: Convective heat transfer(W);

*h*: Convective heat transfer coefficient(W/(m^2^·K));

*A*_*2*_: Effective convective heat transfer area of the wall(m^2^);

*t*_*w*_: Temperature of solid surfaces(K);

*t*_*f*_: Coolant temperature(K);

#### 2.3.3. Verification of the continuum hypothesis and basis for turbulence model selection.

Prior to conducting numerical simulations, this study first evaluated the validity of the continuous medium assumption for fluid flow within microchannels. The rationale for this assumption was primarily demonstrated through the following aspects:

1. In this study, Reynolds numbers (*Re*) far exceed 4000. Even within smooth channels, flow has fully developed into turbulence. Inertial forces dominate over viscous forces, resulting in highly random and chaotic motion of fluid particles.
2. Within fin channels, the fins themselves constitute significant geometric discontinuities. As fluid flows past the fins, flow separation occurs on the leeward side, forming a shear layer. A stagnation zone with high pressure exists at the leading edge of the fin, an acceleration zone on the side, and a low-pressure recirculation zone on the back. This intense pressure gradient and velocity shear ensure the continuous generation and transport of turbulent pulsations throughout the flow field, precluding any possibility of laminar flow.
3. The Knudsen number serves as the gold standard for determining whether a fluid satisfies the continuous medium assumption:

*K*_*n*_ < 0.001: Continuous medium region;

0.001 < *K*_*n*_ < 0.1: Slip flow region;

0.1 < *K*_*n*_ < 10: Transition region;

*K*_*n*_ > 10: Free molecular flow.

As shown in Equation (13), the Knudsen number is defined as the ratio of the mean free path of molecules to a characteristic physical length. In this study, the working fluid is deionized water (liquid). Liquid molecules are densely packed, lacking a “free path” analogous to gases. For liquids, lattice spacing is typically used to approximate the mean free path. The effective diameter or lattice spacing of water molecules is approximately 0.3 nm. Substituting this into Equation (13) yields *K*_*n*_ far below 0.001. This indicates that the fluid can be treated as a continuous medium at the microscopic level. Collisions between fluid molecules and wall molecules at the interface occur far more frequently than interactions between fluid molecules themselves. Consequently, the macroscopic velocity at the wall surface is strictly zero. Micro-scale effects (such as rarefied gas effects) can be neglected in this study.


$$K_{n}=\frac{\gamma }{D_{\text{1}}}$$ (13)

In the formula: *K*_*n*_ denotes the Knudsen number, *γ* represents the average free path of fluid molecules(m), while *D*_*1*_ denotes the feature length(m).

### 2.4. Evaluation criteria

The thermal efficiency of liquid cooling plates is primarily determined by the thermal conductivity of the solid material that constitutes the plate, as well as the convective heat transfer associated with fluid flow through its channels. The analysis of heat and mass transfer within these plates employs established fluid mechanics equations. Typically, these calculations utilize dimensionless fluid parameters, notably the *Re* and the *Nu*. To evaluate thermal performance, key metrics include thermal resistance, temperature standard deviation, friction coefficient, and the heat transfer enhancement factor.

The flow channel dimensions of cold plates are typically characterized by the hydraulic diameter:


$$D=\frac{4A_{c}}{P_{w}}=\frac{4\left(cb\right)}{2\left(c+b\right)}=\frac{2cb}{c+b}$$ (14)

In the formula, *A*_*c*_ is the channel cross-sectional area, *P*_*w*_ is the channel wet perimeter, *D* is the hydraulic diameter, m; *c* is the channel width, m; *b* is the channel depth, m.

The *Re* is used as a variable to compare the *Nu*. The *Re* is defined as follows:


$$Re=\frac{\rho V_{\text{in}}D_{\text{1}}}{\mu }$$ (15)

In the formula, *ρ* represents the fluid density (kg/m^3^), *μ* denotes the dynamic viscosity of the fluid (Pa·s), *D*_*1*_ signifies the feature length (m), and *V*_*in*_ indicates the velocity of the fluid at the inlet of the liquid cooling plate (m/s). The heat transfer characteristics of the fluid within the liquid cooling plate are assessed using the average *Nu*, which is defined as follows:


$$Nu=\frac{hD}{\lambda }$$ (16)

In the formula, *h* is the convective heat transfer coefficient of the liquid cooling plate, (W/(m^2^·K)); *D* is the hydraulic diameter, m; *λ* is the thermal conductivity of the fluid, which varies according to the temperature of the fluid, (W/(m·K)).

The Prandtl number represents the relative magnitude of a fluid’s ability to transfer momentum and heat energy, and is defined as follows:


$$Pr=\frac{\mu \cdot Cp}{\lambda }$$ (17)

In the formula, *μ* represents the dynamic viscosity of the fluid measured in Pascal-seconds (Pa·s). The symbol *λ* denotes the thermal conductivity of the fluid, which varies with temperature and is expressed in watts per meter per Kelvin (W/(m·K)). Additionally, *C*_*p*_ signifies the specific heat capacity of the fluid, measured in joules per kilogram per Kelvin (J/(kg·K)).

The heat transfer coefficient is a critical parameter that quantifies the efficiency of heat transfer between a surface and the adjacent fluid. It represents the amount of heat transferred per unit area per unit time between the surface and the fluid, whether by convection or conduction. The average heat transfer coefficient is defined as follows:


$$h_{\text{1}}=\frac{Q}{A_{m}(T_{W_{\text{1}}}-T_{f_{\text{1}}})}$$ (18)

In the formula, *T*_*w1*_ denotes the average temperature of the solid bottom surface of the flow channel (K); *T*_*f1*_ denotes the average temperature of the fluid (K); *A*_*m*_ denotes the total area of the wetted surface in the flow channel (m^2^).


$$h_{\text{2}}=\frac{Q}{A_{m}(T_{W_{\text{2}}}-T_{f_{\text{2}}})}$$ (19)

In the formula, *T*_*w2*_ represents the average temperature of the wall surface surrounding the flow channel (K), while *T*_*f2*_ denotes the average temperature of the fluid (K). *A*_*m*_ denotes the total area of the wetted surface in the flow channel (m^2^).

Thermal resistance *(R*_*TH*_) quantifies the resistance to heat flow through a material or system. It is defined as:


$$R_{TH}=\frac{T_{max}-T_{\text{in}}}{Q}$$ (20)

**Fig 3:**
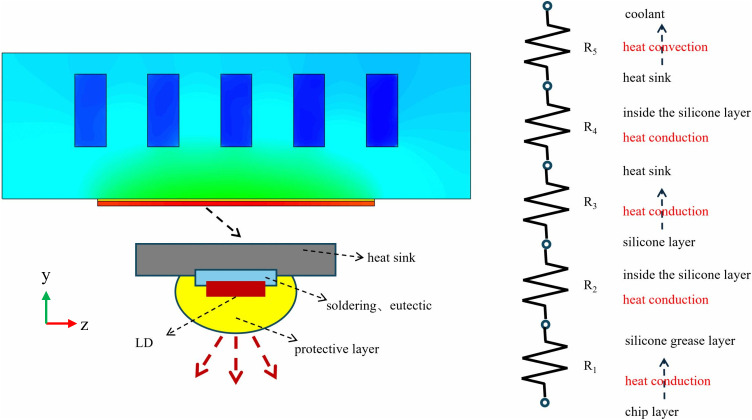
Thermal resistance model layer diagram.

The total thermal resistance of the liquid cooling plate is shown in (Fig 3):

In this formula, *T*_*max*_ represents the maximum temperature of the heat flow surface (°C), while *T*_*in*_ denotes the inlet temperature of the fluid (°C). *Q* indicates the rate of heat transfer through the heat flow surface per unit time (W). The uniformity of the cold plate is characterized by the average absolute temperature difference, denoted as *δ*_*T*_ (°C), defined as:


$$\delta_{T}=\frac{T_{max}-T_{min}}{\text{2}}$$ (21)

In the formula, *T*_*max*_ and *T*_*min*_ represent the maximum and minimum temperatures (°C) of the heat flux surface, respectively. A larger *δ*_*T*_ indicates poorer temperature uniformity of the cold plate and a greater temperature gradient on the surface of electronic components. While evaluating the temperature uniformity of liquid cooling plates, the selected grid nodes are representative; however, the sample size is too small, leading to significant errors. Consequently, this paper utilizes the temperature standard deviation to characterize the temperature uniformity of liquid cooling plates. The temperature standard deviation is defined as follows:


$$\sigma =\sqrt{\frac{{\sum_{t=1}^{n}\left(X-X_{\text{0}}\right)}^{\text{2}}}{n}}$$ (22)

**Fig 4:**
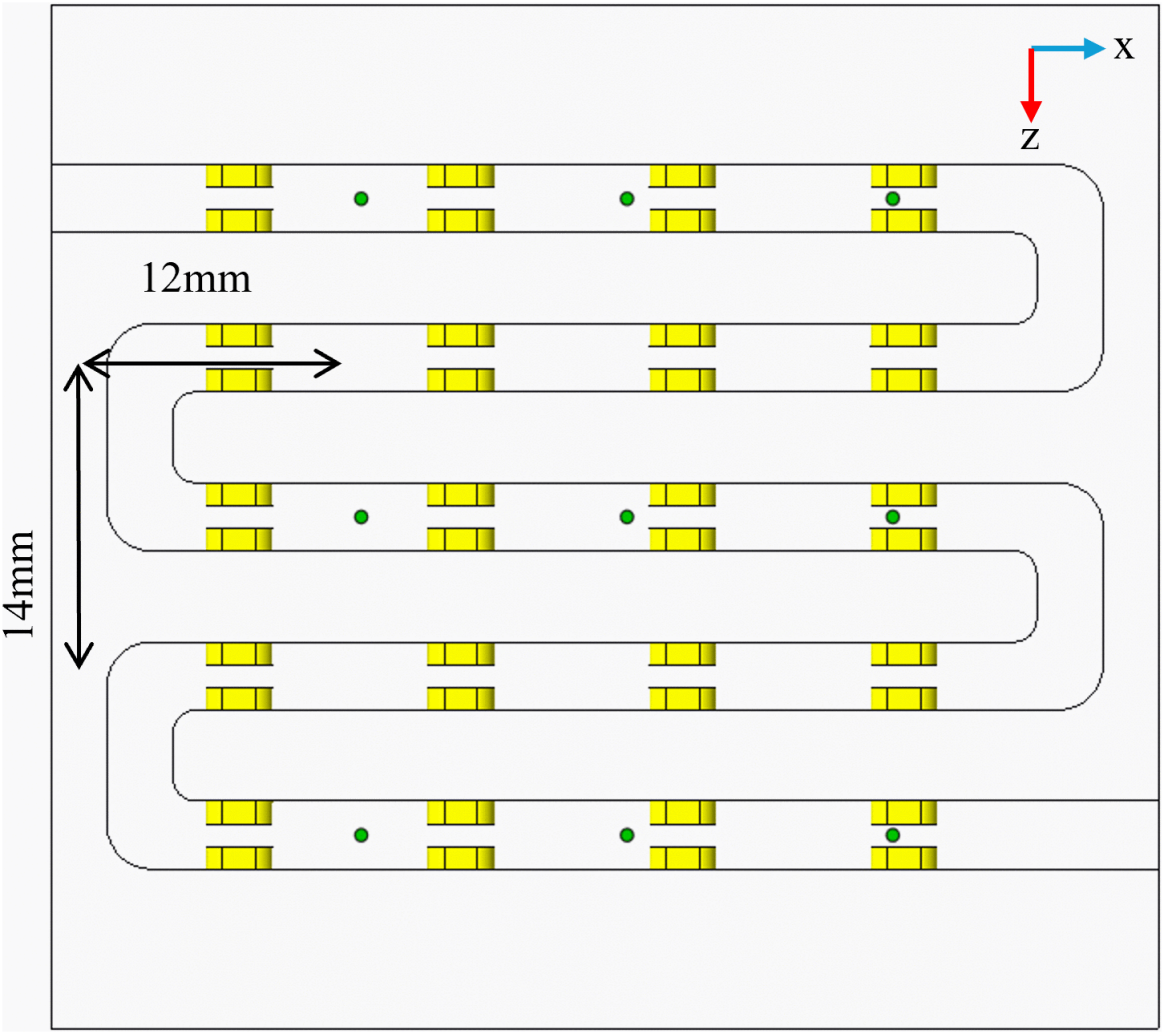
Schematic diagram of grid nine-point selection.

In the formula: *X* is the temperature value on each grid surface (°C), and *X*_*0*_ is the average value of *X* (°C). The error is reduced by increasing the number of samples, which enhances the reference value of the average temperature evaluation. As illustrated in (Fig 4), nine points are captured on the bottom surface of the flow channel in the model, with distances between the points measuring 12 mm and 14 mm, respectively. The wall temperatures at these nine points are used to calculate the temperature standard deviation.

The *f* is defined as the ratio of the wall shear stress to the fluid kinetic energy per unit volume, thereby reflecting the frictional resistance within the MCHS. It is defined as:


$$f=\left(\frac{2D}{\rho v^{\text{2}}}\right)\times \frac{\Delta P}{l}$$ (23)

In the formula, *v* represents the fluid velocity(m/s), *l* represents the perimeter of the flow channel cross-section (m)，*ΔP* represents the pressure drop between the inlet and outlet (kPa).

The thermal enhancement efficiency is a crucial metric that quantifies the improvement in heat transfer performance by comparing the heat transfer coefficients of various cavity micro-channels to those of a smooth micro-channel. This ratio provides insight into the overall effectiveness of different micro-channel designs in enhancing heat transfer, while also reflecting the characteristics of fluid flow within these channels. It is calculated as:


$$PEC=\frac{Nu/Nu_{\text{0}}}{{\left(f/f_{\text{0}}\right)}^{1/3}}$$ (24)

In this study, *Nu* denotes the *Nu* of the investigated micro-channel, while *Nu₀* represents that of the original smooth micro-channel. Similarly, *f* signifies the Fanning friction coefficient of the micro-channel under analysis, and *f₀* denotes the coefficient for the original smooth channel.

### 2.5. Grid independence verification

The modeling work in this study was conducted using the Creo 6.0.0.0 platform, while numerical simulations of the thermal characteristics of the microchannel were performed with the FLOEFD 2020.2 platform. Before commencing numerical simulation experiments, it is crucial to select an appropriate grid size. In simulation contexts, an increase in grid count enhances computational accuracy but concurrently elevates computational costs. Conversely, a reduction in grid count decreases computational accuracy while also reducing computational expenses. Therefore, it is imperative to conduct grid independence tests to determine the optimal grid size. A formula for relative error is formulated as an average metric to assess the adequacy of the grid, defined as follows:


$$e=\frac{\left|M_{\text{2}}-M_{\text{1}}\right|}{M_{\text{1}}}\times 100\%$$ (25)

**Fig 5:**
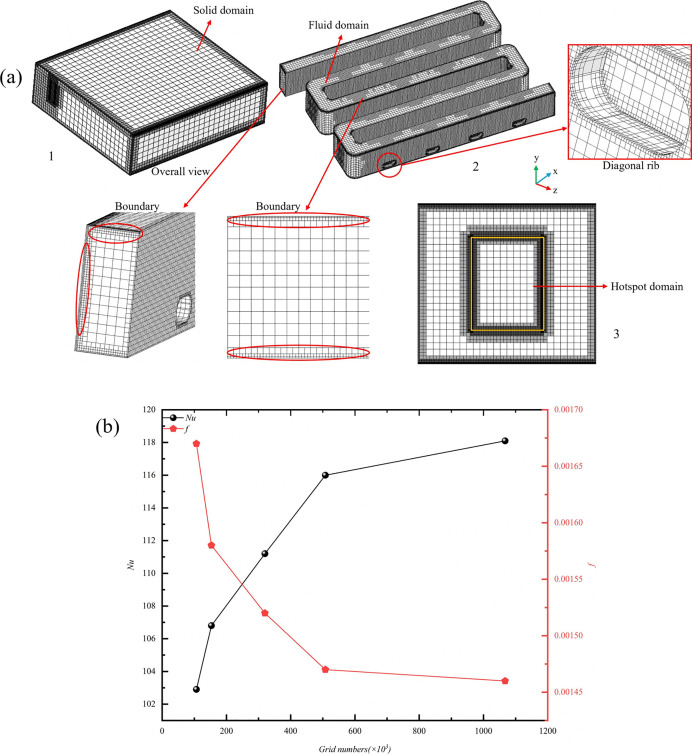
Grid Independence Verification. **(a)** Geometric mesh, **(b)** Verification of grid independence.

Here, *M1* can represent numbers such as the *Nu* and the *f*. *M1* denotes the optimal grid calculation value, while *M2* represents other grid calculation values. By comparing the different grid counts *M1* and *M2*, the value *e* is obtained. The smaller the value of *e*, the higher the accuracy of the grid. In this study, a micro-channel model with rib length *L*_*r*_ = 1.5 mm, rib width *W*_*r*_ = 1 mm, rib height *H*_*r*_ = 1.5 mm, and *Re* = 11353 was selected to investigate the *Nu* and *f* values of the cold plate corresponding to different grid numbers. Using the same grid generation method, five grid sets were created, with mesh counts of 106,603; 153,292; 319,836; 507,868; and 1,067,300. As shown in (Fig 5a), the capsule-shaped fin microchannel was meshed using a tetrahedral grid. In (Fig 5a), 1, 2, and 3 represent the solid mesh of the entire structure, the internal fluid mesh, and the heat source surface mesh. The global mesh for the entire structure is denoted as 1. To ensure computational efficiency, the element size is set to 1 mm. 2 denotes the fluid domain. Considering the minimum fin dimension *L*_*r*_ of 0.5 mm and the fact that this region is critical for flow separation and vortex generation, a fine mesh is necessary to capture flow details. Accordingly, the mesh size in this region ranges from 0.2 mm to 0.5 mm. Adopting a mesh strategy of “refining near boundaries and coarsening in the main flow area” achieves a balance between computational accuracy and cost. Local mesh refinement was applied near the wall surface and on the fins to conform to the wall surface and accurately capture velocity and temperature in the near-wall region, the mesh size was set to 0.2 mm. 3 represents the heat source surface, which serves as the core heat transfer boundary. A local mesh is applied to the solid domain containing the heat source, with the element size refined to 0.2 mm. This ensures node matching at the fluid–solid interface, thereby minimizing numerical interpolation errors across boundaries. (Fig 5b) presents the *Nu* and *f* values obtained from each mesh system. As observed in Table 4, compared to the mesh system with 1,067,300 meshes, when the number of grids reaches 507,868, the relative errors for *Nu* and *f* are 1.8% and 0.7%, respectively, indicating sufficient accuracy. This indicates that the impact of further increasing the number of grids on the simulation results can be considered negligible. Considering both computational accuracy and time cost, the grid system with 507,868 grids is adopted for subsequent simulations.

**Table 4:** Grid independence test (*Re* = 11353).

Grid number	*Nu*	*e* of *Nu*	*f*	*e* of *f*
106603	102.9	12.9%	0.00167	14.4%
153292	106.8	9.6%	0.00158	8.2%
319836	111.2	5.8%	0.00152	4.1%
507868	116	1.8%	0.00147	0.7%
1067300	118.1	–	0.00146	–

### 2.6. Numerical model validation

**Fig 6:**
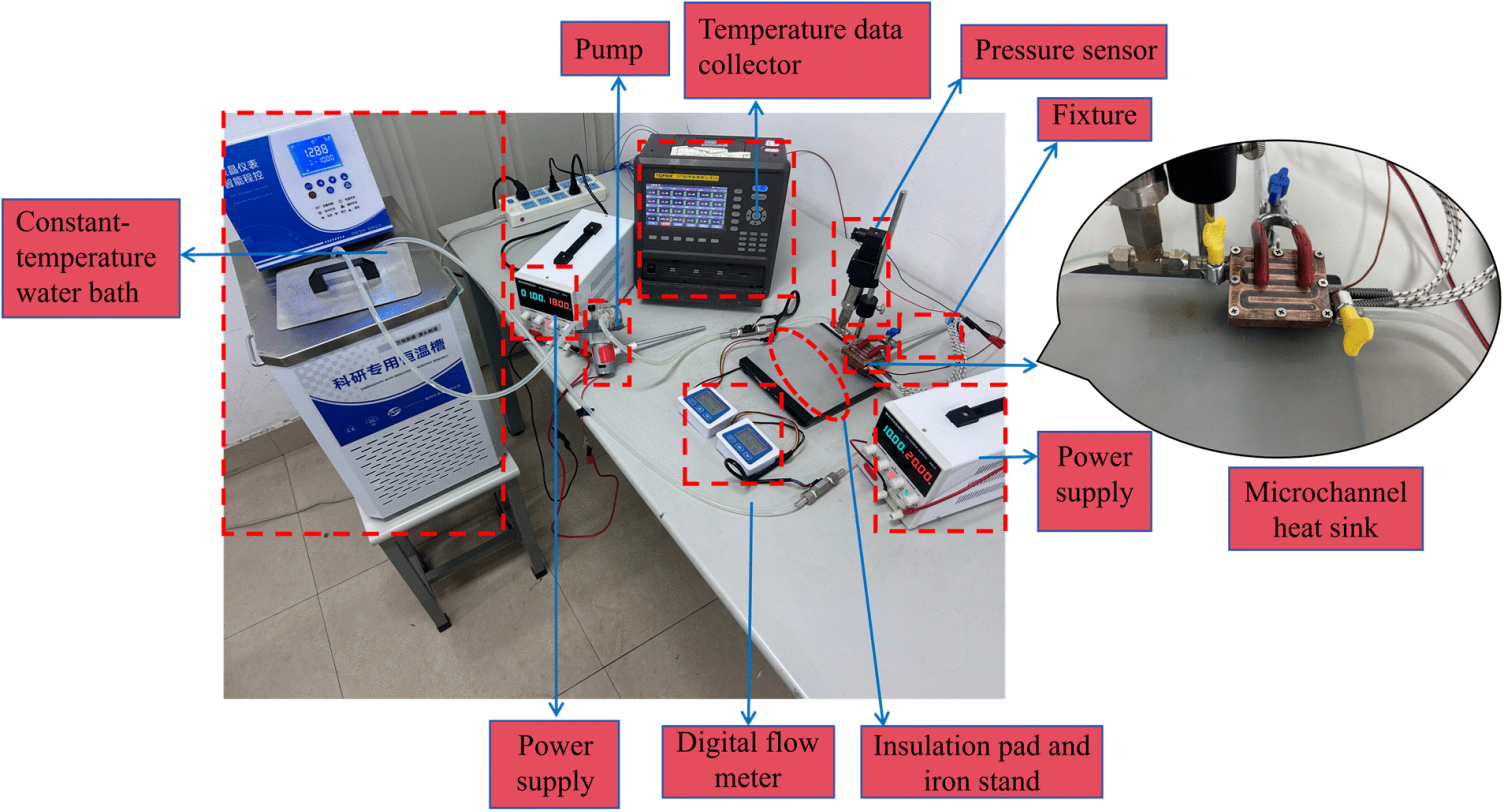
Experimental platform.

**Fig 7:**
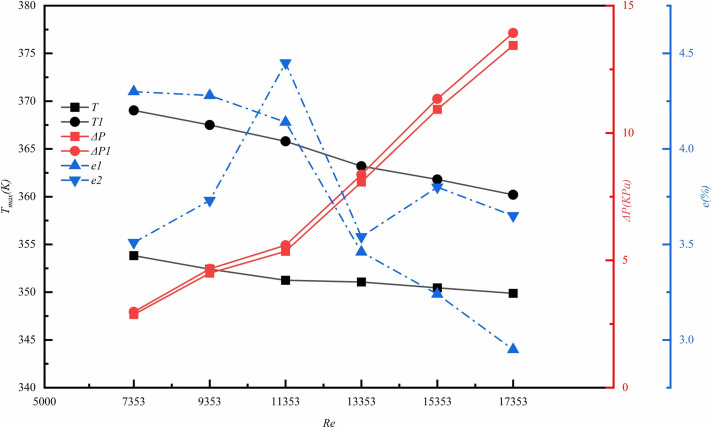
Experimental and numerical simulation error validation.

To validate the accuracy of the model, this study constructed an experimental platform for physical testing. As illustrated in (Fig 6), a physical prototype of the smooth microchannel was fabricated to assess the effects of varying *Re* on the maximum temperature and pressure drop of the heat sink. Furthermore, as depicted in (Fig 7), the maximum deviation in temperature observed in the current numerical simulation occurred at *Re* = 7353, with an error of 4.3%. In contrast, the maximum deviation in pressure drop was noted at *Re* = 11353, with an error of 4.45%. Both errors are below 5%, which indicates that the current method is reliable and that the numerical simulation demonstrates reasonable accuracy. Consequently, it can be effectively utilized for performance prediction and analysis in this study.

## 3. Results and discussion

### 3.1. Preliminary simulation results and analysis of their causes

**Fig 8:**
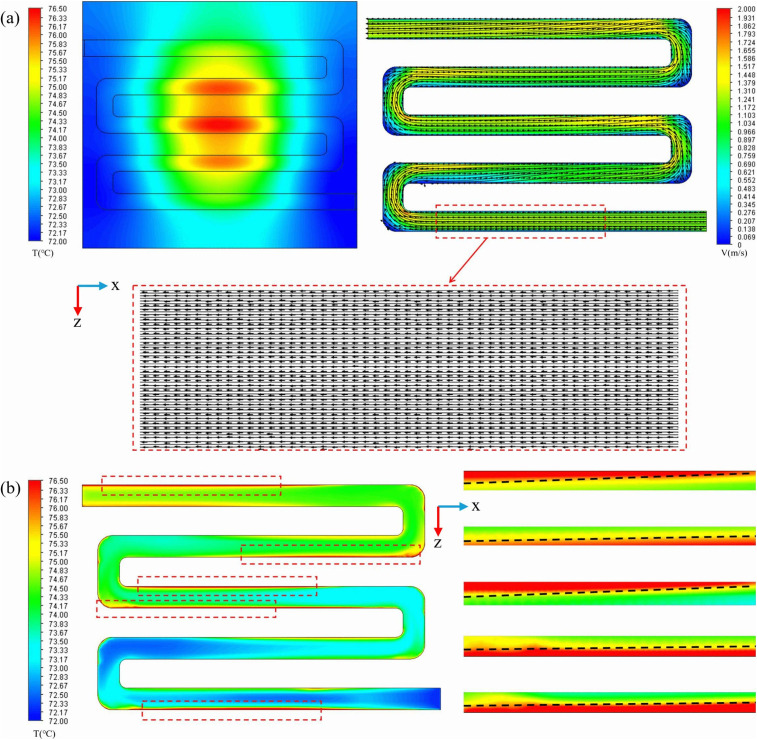
Flow channel temperature and velocity distribution diagram. **(a)** Temperature distribution and flow velocity cloud charts of the smooth channel, **(b)** Thermal boundary.

To establish an optimization baseline for this study and identify the bottlenecks of traditional structures in thermal management, numerical simulations were first conducted on a smooth micro-channel. (Fig 8a) illustrates the temperature distribution at the bottom of the flow channel and the velocity streamline distribution within the channel under turbulent conditions at a Reynolds number of 11353. As shown in (Fig 8a) and (Fig 8b), the fluid flow within the smooth channel remains relatively stable, without forming significant disturbances. Consequently, heat cannot be effectively dissipated and primarily accumulates in the lower region of the chip, resulting in a notable heat accumulation phenomenon. This finding indicates that convective heat transfer in a smooth channel alone is insufficient to meet the cooling requirements of high-power density devices. To address the aforementioned heat accumulation issue, targeted measures for enhancing heat transfer must be implemented.

**Fig 9:**
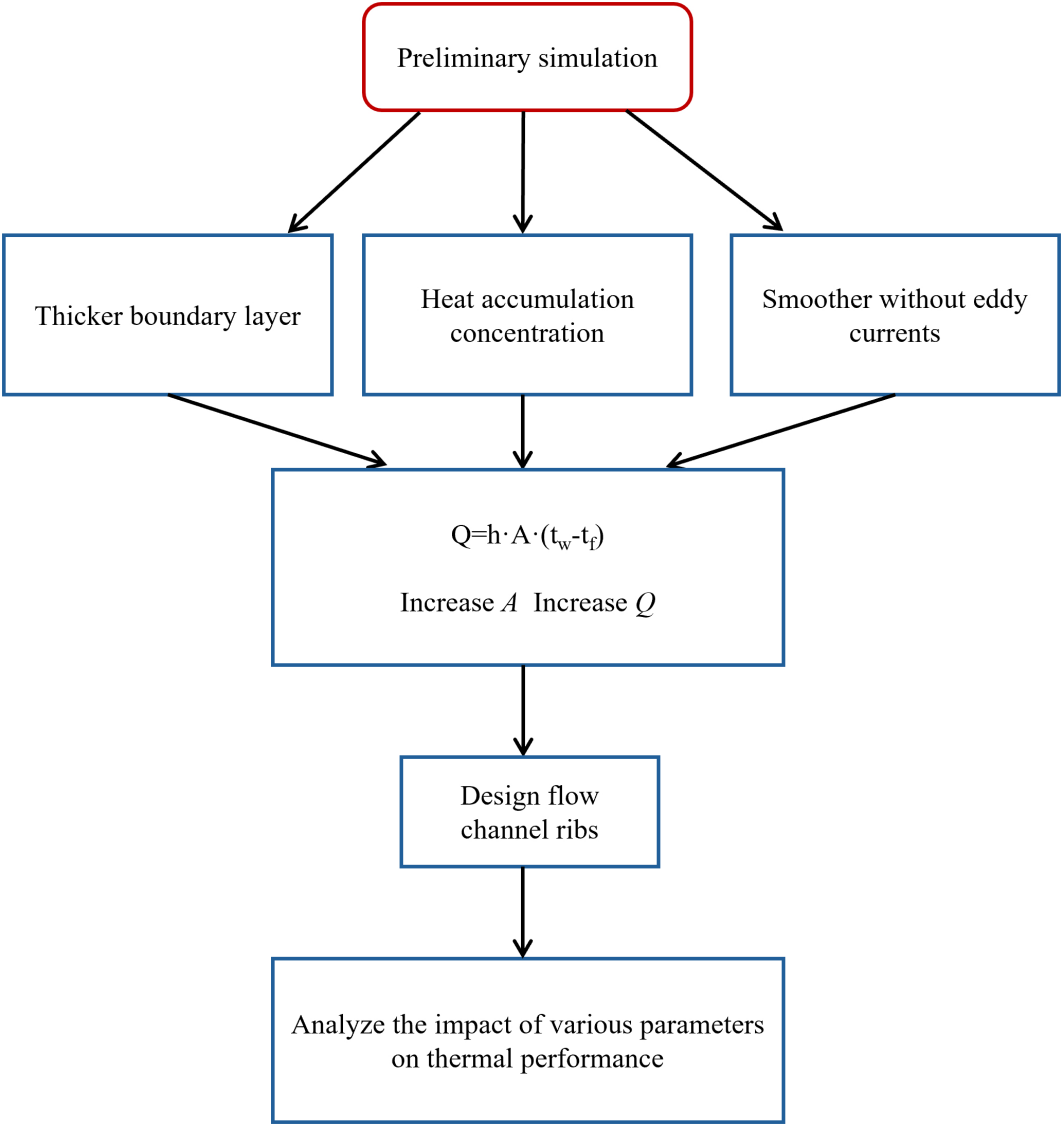
Preliminary simulation analysis diagram.

To clearly elucidate the logical progression from problem identification to strategy formulation, (Fig 9) presents a flowchart of the preliminary simulation analysis. As depicted in (Fig 8b), the primary physical causes of heat concentration are an excessive boundary layer thickness and insufficient vortex disturbance. Based on Newton’s law of cooling, strategies to enhance cooling performance primarily involve reducing boundary layer thickness, increasing fluid velocity, expanding the wall heat transfer area, and selecting thermal interface materials with high thermal conductivity. This study focuses on modifying the internal geometry of the channel by incorporating fin structures to increase the effective heat transfer area. This design induces fluid turbulence, serving as a key solution to mitigate heat accumulation issues. The preliminary simulation results and the analysis of the underlying causes establish a theoretical foundation for the detailed investigation of various rib parameters in the subsequent sections.

### 3.2. The influence of different fin shapes

**Fig 10:**
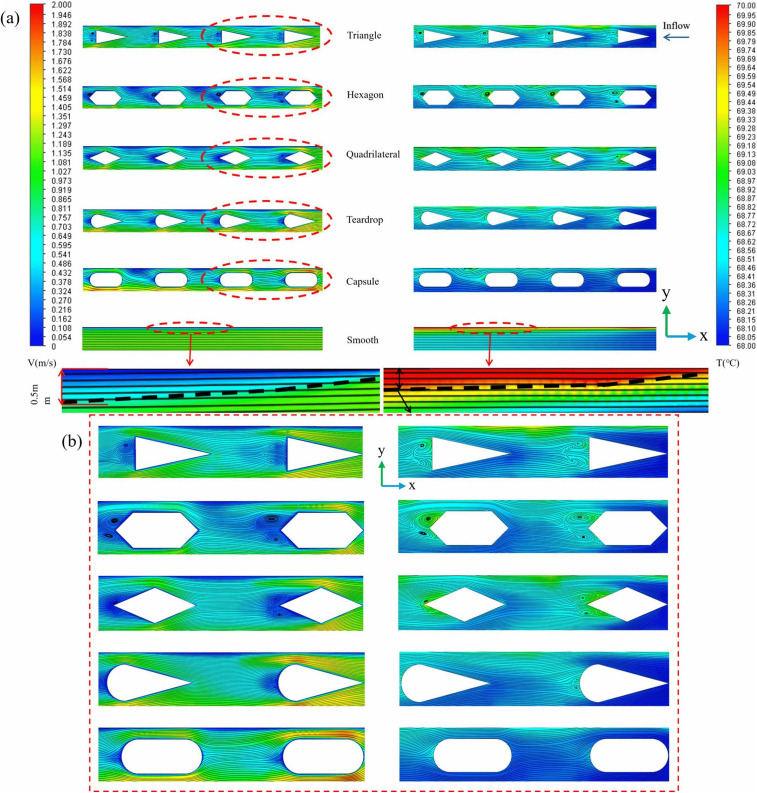
Graphs of different performance parameters. **(a)** Contours of velocity, streamlines, and temperature at the segmented longitudinal sections of *z* = 9.5 mm for different fin shapes at *Re* = 11353, *L*_*r*_ = 2 mm, *W*_*r*_ = 1.2 mm, *H*_*r*_ = 1.5 mm, *L*_*s*_ = 3.5 mm, **(b)** Localized enlarged view.

**Fig 11:**
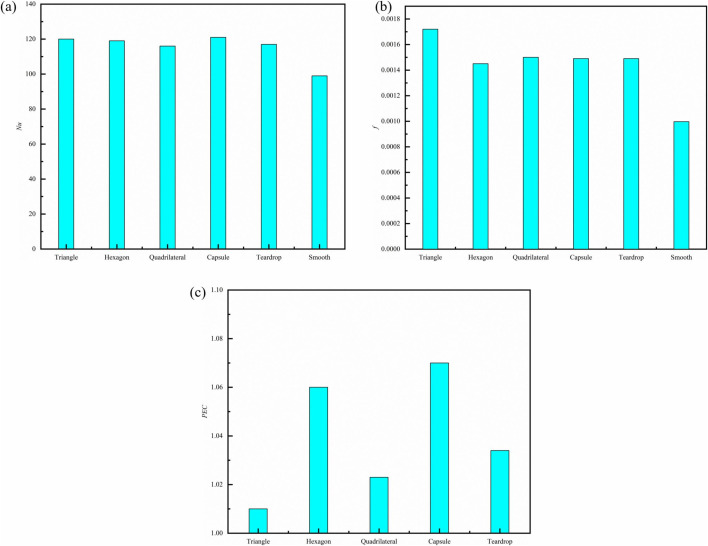
Graphs of different performance parameters. **(a)** *Nu* with fins shape, (b) *f* with fins shape, **(c)** *PEC* with fins shape *Re* = 11353, *L*_*r*_ = 2 mm, *W*_*r*_ = 1.2 mm, *H*_*r*_ = 1.5 mm, *L*_*s*_ = 3.5 mm.

A preliminary investigation into fin selection was conducted. (Fig 10) illustrates the velocity contours, streamlines, and temperature distributions along the longitudinal section at *z* = 9.5 mm. When fluid flows over a solid surface, a narrow region called the boundary layer develops. Viscous effects within this layer reduce the fluid velocity to zero at the wall, creating a significant velocity gradient; this is known as the flow boundary layer. Simultaneously, the temperature difference between the surface and the fluid leads to the formation of an adjacent thin layer exhibiting a steep temperature gradient. This layer is referred to as the thermal boundary layer and constitutes the primary thermal resistance to heat transfer. As depicted in (Fig 10), geometric protrusions of the fin structure disrupt the development of the flow boundary layer [61]. In smooth channels, the thermal boundary layer progressively thickens in the flow direction, forming a substantial thermal resistance layer that leads to heat accumulation at the channel bottom. Fins increase wall roughness, thereby elevating shear stress [61]. Moreover, momentum exchange resulting from flow separation and secondary flow [2] intensifies mechanical energy dissipation, significantly enhancing the mixing between the main flow and the near-wall fluid regions. Triangular fins exhibit the highest friction resistance due to their sharp vertex angles, which cause abrupt bending of the streamline at the leading edge, thereby increasing the likelihood of flow separation [2]. Hexagonal fins generate organized vortex pairs at the trailing edge, enhancing near-wall radial fluid mixing [62]. This effectively thins the thermal boundary layer and elevates the local *Nu*. Quadrilateral fins, characterized by near-right-angle leading edges, are prone to flow separation [2], resulting in multiple end vortices and increased friction resistance. The droplet-shaped fin combines a streamlined leading edge with a tapered trailing edge. Conversely, as shown in (Fig 11)，the capsule-shaped fin achieves the highest *PEC*, signifying an optimal balance between heat transfer enhancement and friction cost [63]. Despite having identical projected areas, the capsule fin offers a larger actual surface area. Crucially, its streamlined leading edge minimizes the initial separation region, reduces flow dead zones, disrupts the thermal boundary layer, and enhances heat transfer, leading to an overall improvement in thermal-hydraulic performance [63]. In summary, since capsule-shaped fins demonstrate the most optimal comprehensive thermohydraulic performance, this study will focus on capsule-shaped fin microchannels for further in-depth investigation.

### 3.3. Comparison of flow field characteristics

**Fig 12:**
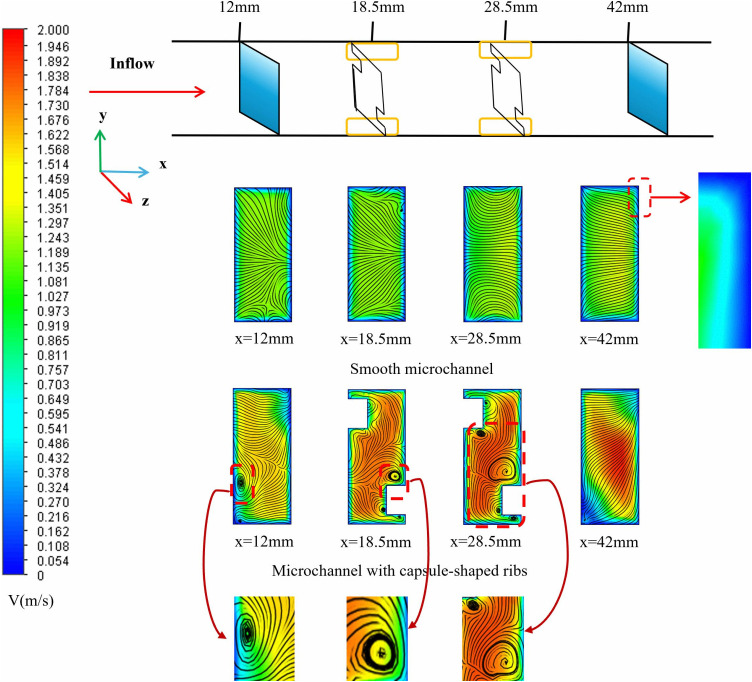
Comparison of the velocity and streamlines contours at different cross sections between the smooth and designed micro-channel at *Re* = 11353.

The impact of capsule-shaped ribs on the micro-channel flow field was examined. (Fig 12) illustrates a comparison of velocity and streamline profiles across different cross-sections for both the smooth micro-channel and the proposed micro-channel at *Re* = 11,353. The dimensions for the capsule-shaped ribs in the designed micro-channel are as follows, *L*_*r*_ = 1.5 mm, *W*_*r*_ = 1 mm, and *H*_*r*_ = 1.5 mm. As depicted in (Fig 12), smooth microchannels exhibit smoother flow due to the absence of fins, making boundary layers prone to form within the flow channels, particularly in the channel corners where a substantial boundary layer develops, leading to heat accumulation in the lower region of the chip. In contrast, in the micro-channel with capsule-shaped ribs, these ribs periodically disturb the fluid flow, generating longitudinal vortices [62]. These vortices vary in size within the channel and effectively inhibit the development of the boundary layer, thereby enhancing heat transfer efficiency. As the fluid approaches the ribs, vortices gradually emerge and expand from the region adjacent to the rib wall toward the center of the channel. Upon passing through the fins, the fluid flow experiences a sudden contraction, which increases the local velocity and enhances the local jet effect. Compared to smooth micro-channels, the incorporation of semi-capsule-shaped fins significantly reduces the thickness of the boundary layer. On one hand, the increase in local velocity facilitates the disruption of the initial boundary layer; on the other hand, the alternating fins periodically disturb the evolution of the boundary layer, ultimately leading to a reduction in its thickness. The observed phenomena reveal that the geometric properties of the ribs have a direct impact on disturbances in flow field intensity and vortex dynamics. Specifically, the longitudinal rib dimension (*L*_*r*_) determines the available space for vortex generation, while the transverse dimension (*W*_*r*_) affects the extent of jet impact. Additionally, the vertical height (*H*_*r*_) is intricately related to the strength of secondary flow [63]. Furthermore, the *L*_*s*_ between staggered fins can modify the periodic behavior of flow separation and reattachment by altering the spatial arrangement of vortex units. Optimizing the shape of the fins could further help balance flow resistance with heat transfer benefits. Given these insights, the present study aims to comprehensively investigate how variations in fin length, width, height, spacing, and geometric shape influence flow and heat transfer characteristics. This research seeks to establish a quantitative relationship between these geometric parameters and thermal-hydraulic performance, thereby providing a foundation for optimizing fin design in heat transfer applications.

### 3.4. The effect of rib length

**Fig 13:**
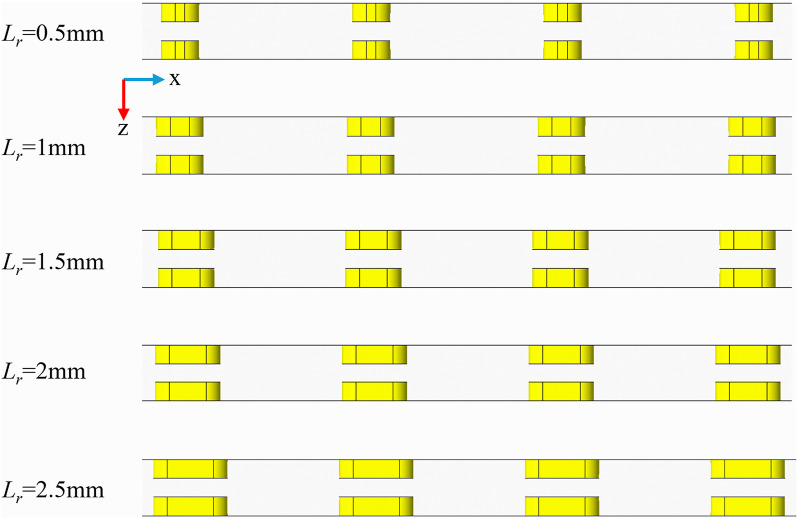
Diagram of flow channel dimensions with different *L*_*r*_ at *Re* = 11353, *W*_*r*_ = 1 mm, *H*_*r*_ = 1.5 mm.

**Fig 14:**
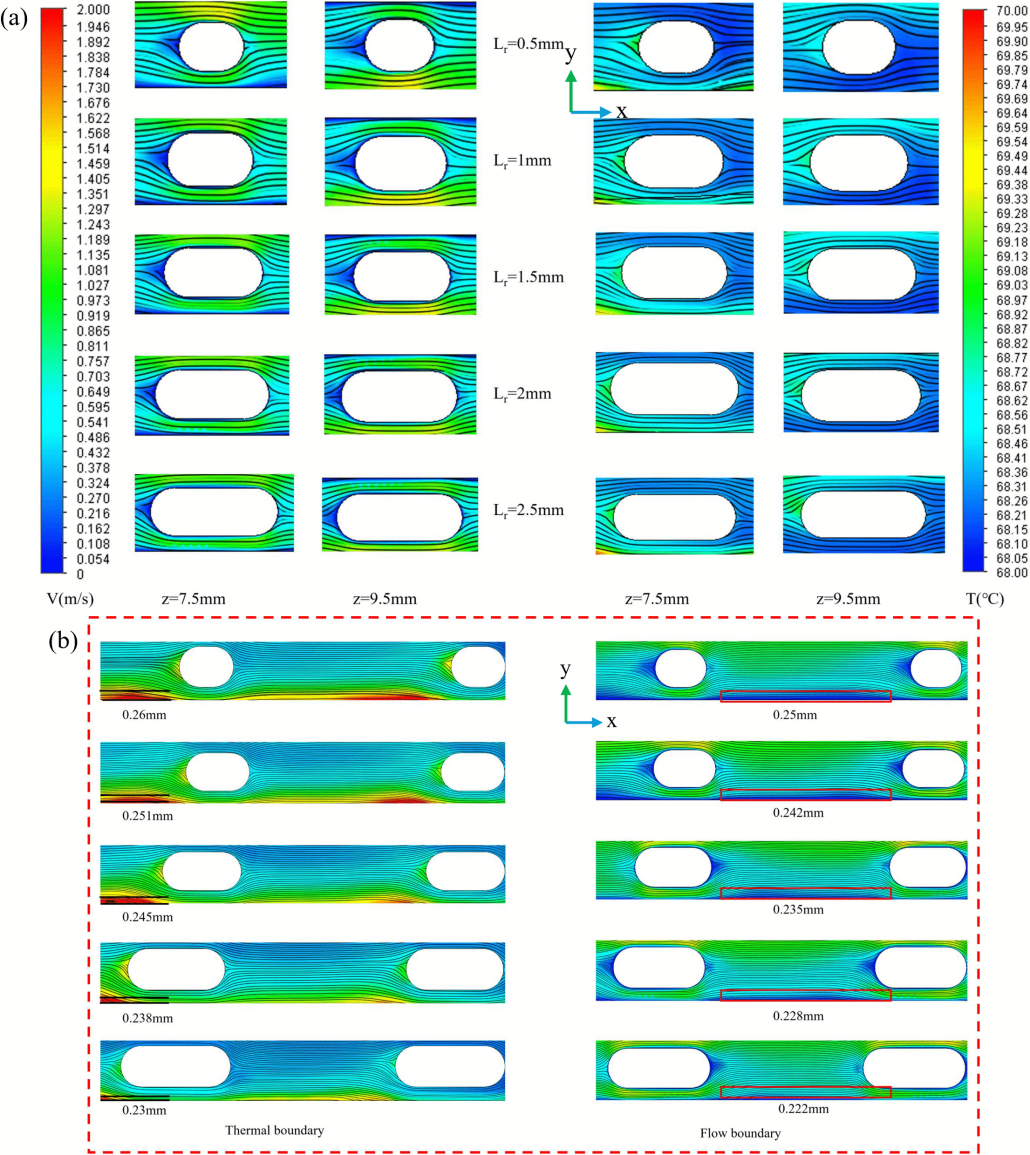
Flow channel temperature and velocity streamline distribution diagram. (a) Contours of velocity, streamlines, and temperature at the segmented longitudinal sections of *z* = 7.5 mm and *z* = 9.5 mm for different *L*_*r*_ at *Re* = 11353, (b) localized enlarged view.

(Fig 13) is the physical model structure diagram. (Fig 14a) illustrates the contour plots of velocity, streamline, and temperature distribution across two longitudinal cross-sections at *Re* = 11353, a rib width of 1 mm, and a rib height of 1.5 mm. These cross-sections are positioned at *z* = 7.5 mm and *z* = 9.5 mm, respectively, and both traverse the center of a rib arrangement within the channel unit along the x-axis. It is evident that for smaller values of *L*_*r*_, the ribs exhibit greater curvature. After fluid separation occurs at the leading edge of the fin, the insufficient lateral length of the fin prevents the separated shear layer from reattaching to the fin wall before reaching the trailing edge. This results in the formation of a broad and unstable wake region on the leeward side of the fin. Although this wake exhibits high turbulence, its extremely low flow velocity leads to poor convective heat transfer capability. Consequently, a distinct low-velocity zone forms behind the fin, which hinders vortex formation. Furthermore, low-speed zones are also present at the front end of the ribs, resulting from flow originating from the preceding rib unit within the channel, which has not completely dissipated. As *L*_*r*_ increases, the strength of the vortex effect intensifies, causing a gradual contraction of the low-speed zones. Changes in the fluid separation point and the wake flow behavior behind the ribs contribute to reducing the size of the low-speed regions. The temperature distribution contour plot depicted in (Fig 14b) correlates with the velocity distribution shown in part (a). With smaller *L*_*r*_ values, a clear stratification of temperature is observed, accompanied by a significant temperature rise at the rear end of the rib, aligning with the low-velocity region noted in part (a). As *L*_*r*_ increases, the enhanced vortex effect promotes fluid mixing and weakens the thermal boundary layer, which improves temperature uniformity and decreases stratification. Notably, optimal heat transfer performance is achieved when *L*_*r*_ is set to 2 mm.

**Fig 15:**
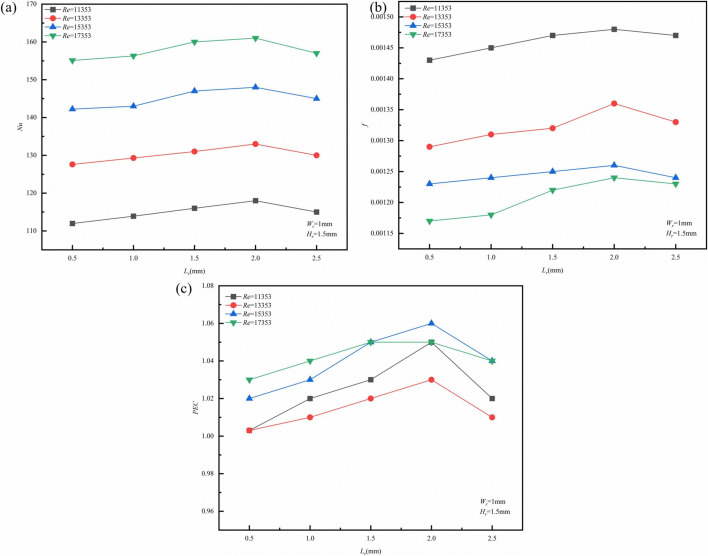
Graphs of different performance parameters. **(a)** *Nu* with *L*_*r*_ at different *Re*, (b) *f* with *L*_*r*_ at different *Re*, **(c)** *PEC* with *L*_*r*_ at different *Re.*

The variation curves for *Nu*, *f*, and *PEC* concerning *L*_*r*_ across different *Re* are illustrated in (Fig 15). As the fin length increases, the *Nu* value show an upward trend. Primarily because longer fins not only expand the effective heat transfer area but, more importantly, enhance the ‘flow-guiding effect.’ Longer fins compel the fluid to deflect over a greater distance within the flow channel, thereby intensifying the fluid’s impact on the side walls.This phenomenon leads to a reduction in the thickness of the thermal boundary layer, resulting in greater temperature uniformity and improved thermal performance of the liquid-cooled plate [32,33]. Additionally, as *Re* increases, the thermal performance of micro-channels improves, facilitating faster flow and a thinner boundary layer. The friction factor f initially rises with longer *L*_*r*_ but subsequently declines as *L*_*r*_ continues to increase. Initially, longer fin lengths exacerbate fluid flow separation, elevate local turbulence intensity, wall shear stress, and increase friction resistance. However, as the fin length is further extended, the flow tends to stabilize into an attached flow state, reducing turbulence in the wake region and minimizing friction energy loss. The decreased curvature of the fins may enhance the pressure distribution within the flow channel, alleviating the adverse effects caused by secondary flow. Additionally, higher values of *Re* correlate with lower *f*, a phenomenon linked to the diminishing influence of viscous forces and the increased turbulent mixing primarily governed by inertial forces. Both factors collectively reduce energy loss per unit of flow length. The *PEC* increases progressively, reflecting a balanced interplay between improvements in heat transfer and resistance losses. While the *f* peaks at a length ratio (*L*_*r*_) of 2 mm, the continuous increase in the *Nu* predominantly drives the overall enhancement in performance. Lengthening the fins increases the disruption of the thermal boundary layer, thereby improving the coupling efficiency of heat flux density with the velocity field, which leads to a higher heat transfer efficiency per unit of pressure drop.

### 3.5. The effect of rib width

**Fig 16:**
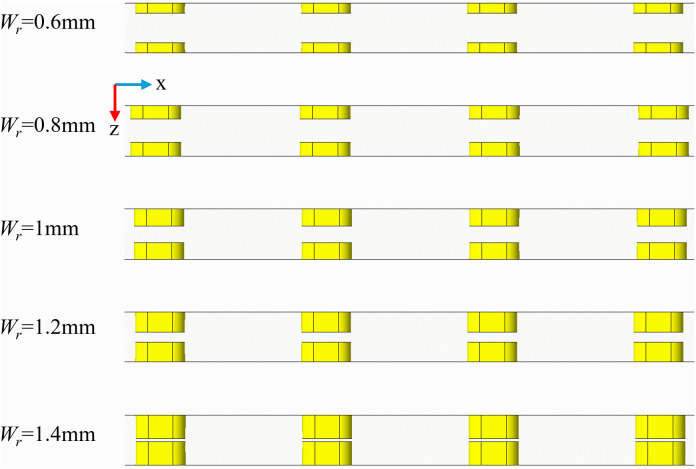
Diagram of flow channel dimensions with different *W*_*r*_ at *Re* = 11353, *L*_*r*_ = 1.5 mm, *H*_*r*_ = 1.5 mm, *L*_*s*_ = 6.5 mm.

**Fig 17:**
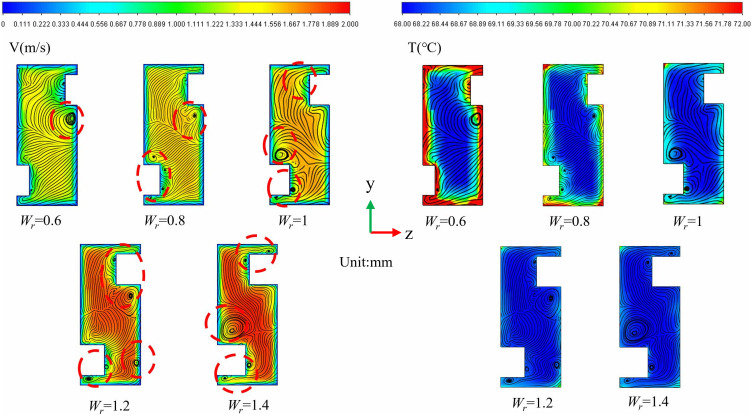
Contours of velocity, streamlines, and temperature at the micro-channel cross section of *x* = 18.5 mm for different *W*_*r*_ at *Re* = 11353.

(Fig 16) is a schematic diagram of the physical model structure. (Fig 17) illustrates the distribution maps of velocity, streamlines, and temperature at the *x* = 18.5 mm cross-section under identical conditions of *Re* = 11353, *L*_*r*_ = 1.5 mm, and *H*_*r*_ = 1.5 mm for varying *W*_*r*_. In (Fig 17), it is observed that when *W*_*r*_ is small, the ribs exert a negligible influence on the flow field, resulting in minimal vortex formation. As *W*_*r*_ increases, the regions of vortex activity expand, and their influence becomes more pronounced. The alternating arrangement of the ribs leads to a diagonal distribution of the vortices. A larger *W*_*r*_ enhances the jet effect, according to the mass conservation equation, maintaining a constant mass flow rate while increasing *W*_*r*_ reduces the flow area, which in turn elevates the local flow velocity. This increase in local velocity is directly proportional to the cross-sectional contraction ratio. Consequently, as *W*_*r*_ increases, the fluid within the fin gaps accelerates, forming high-velocity jets. This high-momentum fluid significantly reduces the thickness of the thermal boundary layer. The velocity distribution depicted in (Fig 17) aligns with the temperature distribution presented in (Fig 17), where regions of higher velocity correspond to lower temperature zones. The ribs positioned on the bottom and top walls of the micro-channel contribute to increased turbulence intensity, thereby facilitating- thorough mixing of hot and cold flows.

**Fig 18:**
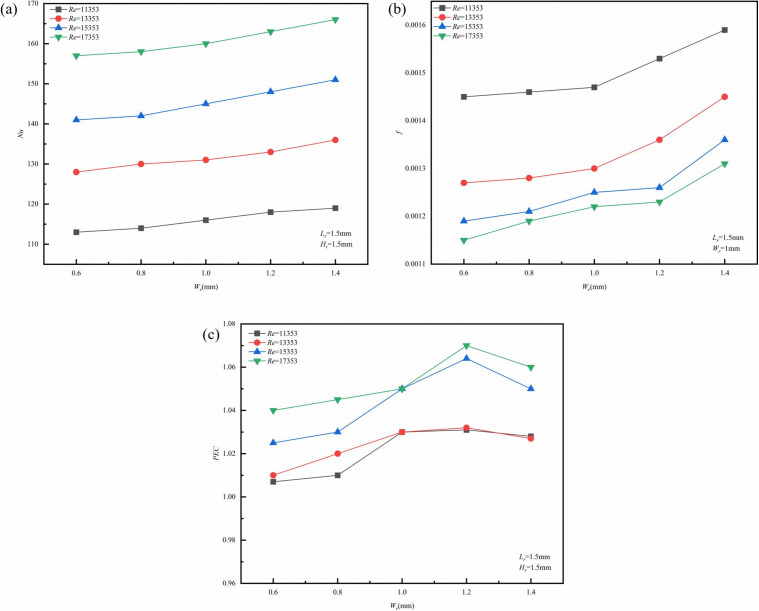
Graphs of different performance parameters. **(a)** *Nu* with *W*_*r*_ at different *Re*, (b) *f* with *W*_*r*_ at different *Re*, **(c)** *PEC* with *W*_*r*_ at different *Re*.

To examine the influence of *W*_*r*_ on the thermal-hydraulic performance of micro-channels, curves representing the *Nu*, *f*, and *PEC* in relation to *W*_*r*_ were plotted at various *Re*, as illustrated in (Fig 18a). The analyzed *Re* range was between 11,353 and 17,353, with *Nu* exhibiting an upward trend as *W*_*r*_ increases. This observation can be attributed to the enhancement of local jet effects and vortex activity caused by a larger *W*_*r*_, leading to a reduced thermal boundary layer within the channel, improved temperature uniformity, and enhanced thermal efficiency. The relatively modest increase in *Nu* is attributed to the presence of low-velocity zones located at the rear of the ribs, which somewhat diminishes the overall heat transfer capability. Furthermore, as *Re* increases, the thermal performance of the micro-channel tends to improve, resulting in a thinner thermal boundary layer and elevated velocities. According to (Fig 18b), with an increase in *W*_*r*_, there is a gradual rise in *f*, accompanied by an increased growth rate. This phenomenon is attributed to the increased local flow velocity. According to Bernoulli’s principle, the *ΔP* is proportional to the square of the flow velocity. Thus, while the increase in velocity induced by *W*_*r*_ enhances heat transfer linearly, it simultaneously raises flow resistance. As depicted in Figs 18a and b, the growth rate of *f* is significantly higher than that of *Nu*, suggesting that the pump power loss surpasses the gain in heat transfer. (Fig 18c) illustrates the relationship curves of *PEC* concerning *W*_*r*_ across different *Re*. As *W*_*r*_ escalates, both *Nu* and *f* experience an increase, with the rise in heat transfer outperforming the flow resistance, resulting in enhanced overall performance and an increase in *PEC*.

### 3.6. The effect of rib height-

**Fig 19:**
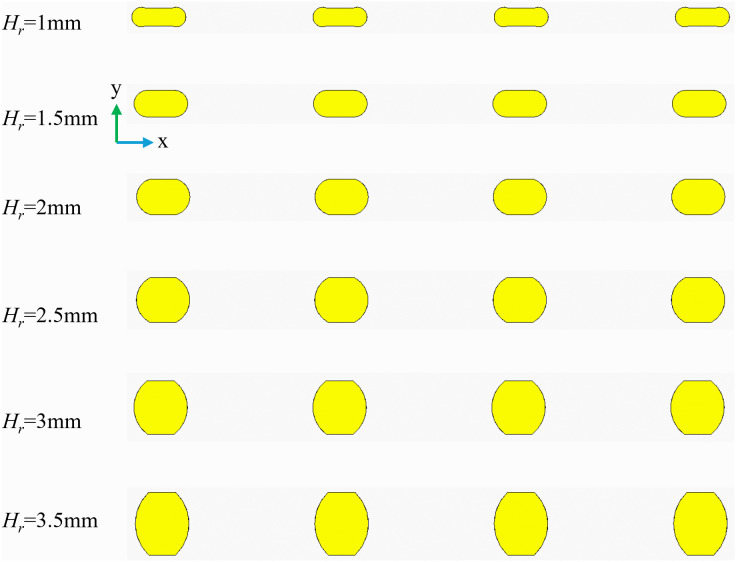
Diagram of flow channel dimensions with different *H*_*r*_ at *Re* = 11353, *L*_*r*_ = 1.5 mm, *W*_*r*_ = 1 mm, *L*_*s*_ = 6.5 mm.

**Fig 20:**
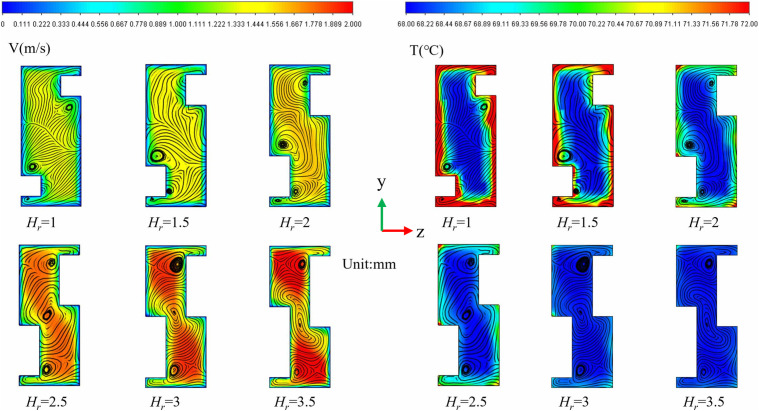
Contours of velocity, streamlines, and temperature at the micro-channel cross section of- *x* = 18.5 mm for different *H*_*r*_ at *Re* = 11353.

**Fig 21:**
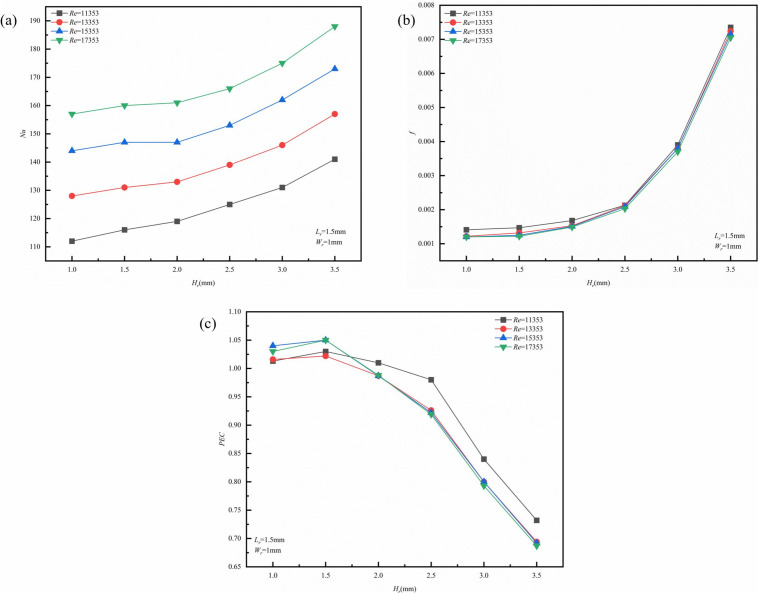
Graphs of different performance parameters. **(a)** *Nu* with *H*_*r*_ at different *Re*, (b) *f* with *H*_*r*_ at different *Re*, **(c)** *PEC* with *H*_*r*_ at different *Re*.

(Fig 19) is the physical model structure diagram. Under conditions of *L*_*r*_ = 1.5 mm, *W*_*r*_ = 1 mm, and *Re* = 11,353, the impact of *H*_*r*_ on the thermal-hydraulic performance of micro-channels was investigated. Fig 18 presents contour plots that illustrate the velocity, streamline, and temperature distributions at the cross-section located at *x* = 18.5 mm. As depicted in (Fig 20), *H*_*r*_ induces vortices within the flow channel. With an increase in *H*_*r*_, these vortices tend to concentrate in two specific regions: the base of the upper rib and the top of the lower rib. This observation suggests that an increase in *H*_*r*_ elevates the channel blockage ratio, compelling the fluid to accelerate through the narrowed cross-section. According to the principle of conservation of mass, the local core flow velocity increases significantly, converting static pressure into dynamic pressure. This enhancement in kinetic energy facilitates the formation of larger-scale horseshoe vortices at the rib base and shear layer separation at the rib top. The expanded vortex area is not merely a geometric scaling; rather, it results from the increased dissipation of turbulent kinetic energy (TKE), which promotes vigorous mixing between the near-wall hot fluid and the core cold fluid. Furthermore, (Fig 20) also demonstrates that the thermal boundary layer becomes progressively thinner, and as *H*_*r*_ increases, the efficiency of heat transfer improves. This enhancement is primarily due to the increase in flow velocity and the amplification of disturbance effects as *H*_*r*_ rises. (Fig 21) illustrates the variations of *Nu*, *f*, and *PEC* concerning *H*_*r*_ across different *Re* values. As shown in (Fig 21a), the growth rate of *Nu* consistently increases with an increase in *H*_*r*_. This phenomenon can be attributed to the reduction of the low-velocity zone caused by a larger *H*_*r*_, which enhances fluid mixing and intensifies vortex formation. Similarly, the growth rate of f exhibits a gradual increase with higher *H*_*r*_, which is associated with increased viscous stress and an overall rise in mechanical energy loss. A notable drop is observed at *H*_*r*_ = 2.5 mm, which can be largely attributed to the significant increase in *f* at this specific *H*_*r*_, as depicted in (Fig 21b). As shown in (Fig 21c), the *PEC* initially increases but then decreases with increasing *H*_*r*_. This trend primarily occurs because, within the *H*_*r*_ range of 1.5 mm to 2 mm, the enhancements in heat transfer efficiency outweigh the increases in flow resistance, making heat transfer the principal contributor to overall performance. Conversely, within the *H*_*r*_ interval of 2 mm to 3.5 mm, the advantages gained from heat transfer diminish relative to the increasing flow resistance, leading to a reduction in *PEC*.

### 3.7. The effect of rib spacing

**Fig 22:**
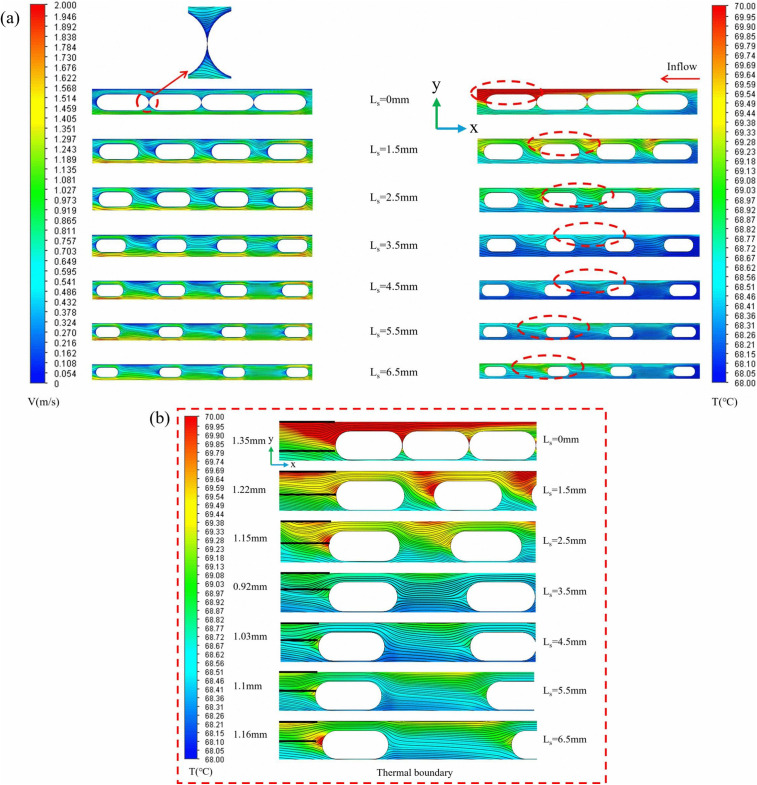
Flow channel temperature and velocity streamline distribution diagram. **(a)** Contours of velocity, streamlines, and temperature at the segmented longitudinal sections of *z* = 9.5 mm for different *L*_*s*_ at *Re* = 11353, *L*_*r*_ = 2 mm, *W*_*r*_ = 1.2 mm, *H*_*r*_ = 1.5 mm, **(b)** Localized enlarged view.

**Fig 23:**
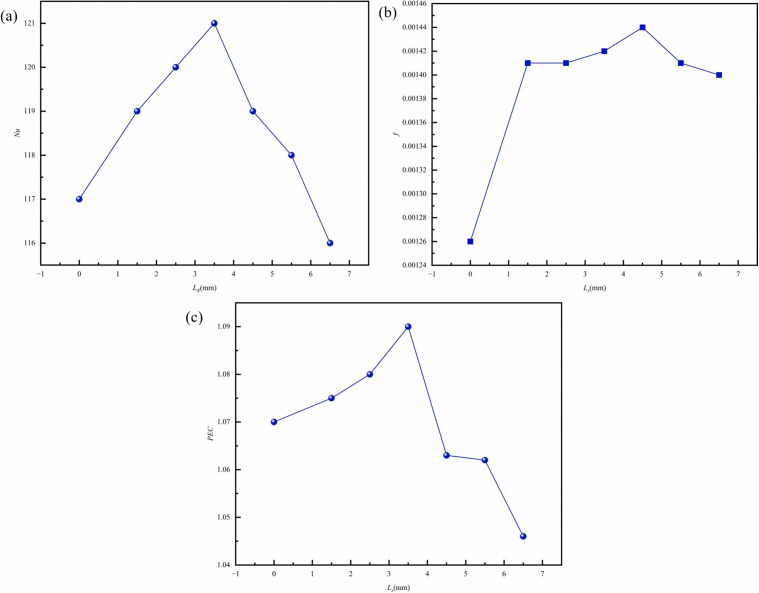
Graphs of different performance parameters. **(a)** *Nu* with *L*_*s*_ at different *Re*, (b) *f* with *L*_*s*_ at different *Re*, **(c)** *PEC* with *L*_*s*_ at different *Re*.

Based on the analysis presented in Sections 3.4, 3.5, and 3.6, the optimal dimensions for the micro-channel model have been determined to be 2.0 mm, 1.2 mm, and 1.5 mm. To further enhance performance, the impact of *L*_*s*_ on thermal-hydraulic performance is examined for ribs with *L*_*r*_ = 2.0 mm, *W*_*r*_ = 1.2 mm, *H*_*r*_ = 1.5 mm, and *Re* = 11,353. The investigated *L*_*s*_ values are 0, 1.5, 2.5, 3.5, 4.5, 5.5, and 6.5 mm.

(Fig 22) shows contours of velocity, streamlines, and temperature at the longitudinal section *z* = 9.5 mm for different *L*_*s*_. The figure clearly shows that when the fin spacing is 0 mm, a continuous solid structure forms between the fins. As the coolant flows through the fins, the flow boundary layers of adjacent fins rapidly merge, resulting in a skimming flow regime. When *L*_*s*_ is significantly smaller than the reattachment length of the separated shear layer, the fluid glides over the rib crests without penetrating the gap. The stable vortices trapped within these gaps effectively insulate the heat transfer surface from the main coolant flow, creating thermal stagnation zones. Consequently, the effective convective heat transfer area is reduced to merely the top surface of the ribs. This phenomenon significantly reduces the convective heat transfer coefficient and diminishes the efficiency of heat transfer from the fin surface to the fluid. As seen in (Fig 22a) (*L*_*s*_ = 0 mm), stagnant zones form at rib junctions, reducing the effective heat transfer area. At the optimal spacing (*Ls* = 3.5 mm), fluid distributes uniformly between ribs, maximizing surface cont act area while minimizing flow separation [2]. On the right side of (Fig 22a), it can be observed that when the spacing *L*_*s*_ is 0 mm, the heat near the fins that are distant from the inlet fluid cannot be effectively dissipated, leading to increased local temperature gradients and the formation of heat accumulation, which further deteriorates heat dissipation performance. (Fig 23) demonstrates that the *Nu* initially increases and then decreases with increasing spacing, while the *f* also follows a similar trend, gradually smoothing out. At *L*_*s*_ = 3.5 mm, the *PEC* reaches its maximum value, indicating optimal thermohydraulic performance.

## 4. Neural networks and genetic algorithms for multi-objective parameter optimization

### 4.1. Description of multi-objective optimization problems

As discussed in the previous section, the dimensions of the rib, *L*_*r*_, *W*_*r*_, *H*_*r*_, and *L*_*s*_, play a crucial role in influencing the hydraulic and thermal efficiency of the channel. However, these individual studies do not identify the global optimum across the entire design space. Therefore, multi-objective optimization is employed to achieve the optimal overall performance. Under the condition of *Re* = 11353, two objective functions, *J1*(*Nu*/*Nu*_*0*_) and *J2*(*f*/*f*_*0*_), have been selected, with the goal of enhancing *J1* while minimizing *J2*. These two optimization functions illustrate the improvement in hydraulic and thermal efficiency of the designed micro-channel compared to the original smooth channel. Based on the simulation results, the parameters *L*_*r*_, *W*_*r*_, *H*_*r*_, and *L*_*s*_ are established at (0.5–2.5 mm), (0.6–1.4 mm), (1–3.5 mm), and (0–6.5 mm), respectively. Consequently, the multi-objective optimization problem is thus defined as finding the optimal combination (*L*_*r*_, *W*_*r*_, *H*_*r*_, *L*_*s*_) within these ranges to maximize overall micro-channel performance.

### 4.2. Combination of neural network algorithms and genetic algorithms

**Fig 24:**
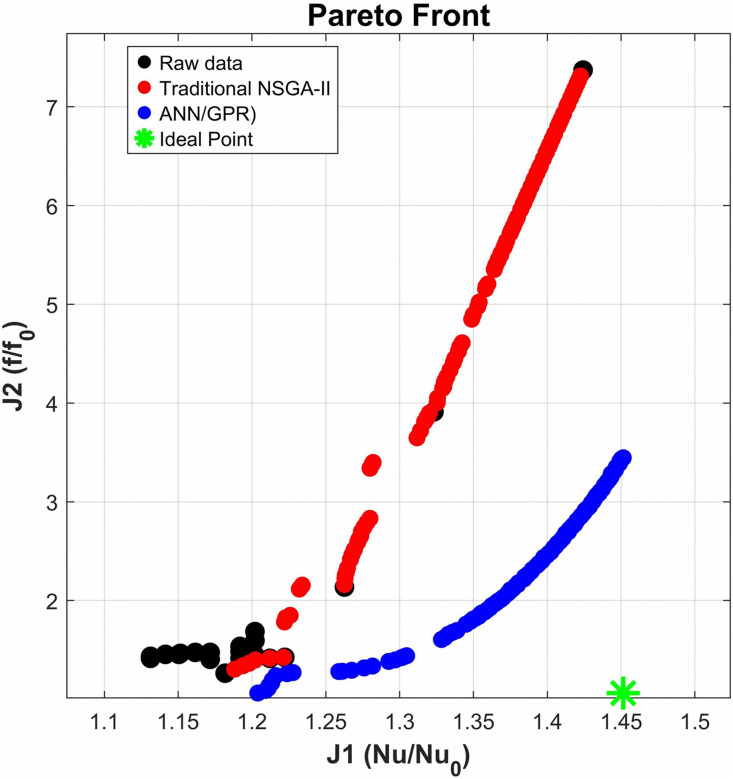
Comparison of Pareto Fronts between traditional NSGA-II and hybrid GPR-DNN enhanced NSGA-II.

**Fig 25:**
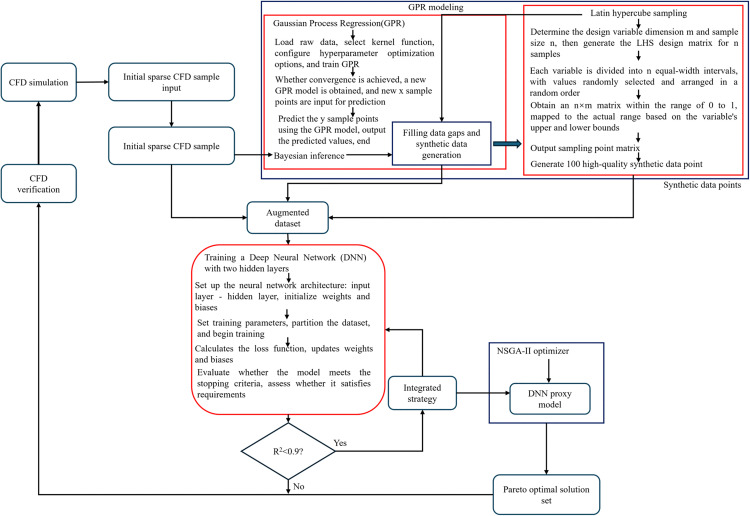
Schematic diagram of a multi-objective optimization Framework for microchannel heat sink based on GPR data augmentation and DNN ensemble learning.

Microchannel heat sinks exhibit complex flow separation, vortex evolution, and boundary layer reconstruction within their structures, revealing highly nonlinear and nonconvex mapping relationships between geometric parameters and thermofluid performance. Traditional response surface methods typically rely on second-order polynomial assumptions, which introduce inherent structural rigidity that can lead to underfitting when addressing high-dimensional complex surfaces. This rigidity often smooths out local optimal peaks in the design space. Conversely, while deep neural networks possess universal function approximation capabilities, the use of sparse small-sample data, coupled with high CFD computational costs, can easily result in model overfitting. To effectively balance model generalization and fitting accuracy within limited computational resources, this study does not rely solely on traditional algorithms. Instead, it constructs a deep learning optimization framework that utilizes Gaussian Process Regression (GPR)-enhanced data augmentation. (Fig 24) illustrates the comparison of the Pareto front between the traditional NSGA-II and the ANN/GPR-enhanced method proposed in this paper. The ANN/GPR method achieves broader coverage of the Pareto frontier and attains higher heat transfer performance at the same flow resistance level, approaching the ideal point more closely. This indicates that the traditional method, which relies on a limited number of raw samples and simple interpolation approximations, results in a relatively concentrated solution set that fails to fully explore the performance boundaries of the design space. Consequently, it is prone to local optimization, whereas the deep learning model successfully uncovers a design space with superior performance. In summary, the algorithmic framework is illustrated in (Fig 25), with the innovative aspects highlighted in red. Additionally, as shown in Table 5, we compared the traditional NSGA-II algorithm with the algorithm developed in this study and summarized the advantages of the latter.

**Table 5:** Technical comparison between hybrid GPR-DNN enhanced NSGA-II and the traditional NSGA-II.

Algorithm	Traditional NSGA-II	Hybrid GPR-DNN enhanced NSGA-II	Advantage
Data preprocessing and augmentation	Directly training surrogate models using limited CFD samples without systematic data augmentation	GPR Data Augmentation: Leveraging the probabilistic nature of Gaussian Process Regression (GPR) to generate dense synthetic datasets from sparse CFD data	Addressing the Small Sample Problem: GPR acts as a “generator” to fill gaps in the design space, enabling deep neural networks to train on large (synthetic) datasets while avoiding costly additional CFD computations
Feature Engineering	The model only has the original design variables and must learn the nonlinear relationships between them on its own.	By incorporating interaction features and quadratic terms, an expanded feature set is constructed to explicitly represent the physical geometric coupling relationships within the model.	Physical Perception Capability: Through predefined interaction items, the model can more accurately capture nonlinear coupling effects in fluid dynamics, enhancing its generalization ability.
Proxy Model Architecture	Shallow networks: Typically single-hidden-layer BP networks or polynomial response surfaces (RSM), with limited fitting capability.	Deep ANN: Dual Hidden Layer Deep ANN + Dynamic Ensemble Strategy	Capturing High-Order Nonlinearities: Deep architectures can capture complex response surface discontinuities caused by turbulent flow fields (such as secondary flows and vortex breakup), with integrated strategies enhancing robustness.
Optimality Efficiency	Inefficient/Prone to Local Optima: Due to insufficient accuracy of the proxy model, GAs often converge to spurious mathematical extrema points.	High-Fidelity Global Optimization: High-precision hybrid surrogate models “smooth” the design space, guiding NSGA-II to rapidly pinpoint true physical optimal solutions.	The cost-benefit ratio is exceptionally high: achieving optimization accuracy typically requiring hundreds of calculations with minimal computational fluid dynamics (CFD) computational effort.
Decision-making mechanism	Manual Selection/Weighting: Typically involves subjectively selecting a point on the Pareto frontier.	TOPSIS Decision-Making: An integrated multi-attribute decision method that objectively selects the “best compromise solution” based on Euclidean distance.	Provide an objective, systematic optimization mechanism to enhance engineering practicality and avoid the influence of subjective preferences.

(Fig 26) illustrates the NSGA-II implementation workflow. Neural network algorithms and genetic algorithms are biologically inspired computational techniques [64–66]. Initially, the neural network algorithm is employed to train the provided samples, followed by a series of learning and training phases until the target accuracy is achieved. This trained ANN model is then used to predict the system outputs. The predicted data samples are subsequently reintroduced into the trained neural network model to validate the model’s predictions. The resulting function of the accurate prediction model is integrated into the genetic algorithm model as the objective function, while the combinations of sample input parameters serve as the targets for optimization. Through iterative calculations performed by the genetic algorithm, the most suitable parameter combinations are ultimately identified, thereby concluding the multi-objective parameter optimization process. Artificial Neural Networks (ANN) have been successfully employed to predict and optimize the thermal performance of heat exchangers with high accuracy [67–69]. In this study, an ANN serves as the surrogate model for training and utilization. Regarding the dataset, 23 sets of base samples were generated via numerical simulation, and an additional 100 new samples were produced through Latin hypercube sampling. The 23 base samples were subsequently used to develop two regression models, *J1* and *J2*, employing Gaussian Process Regression. These trained regression models were then applied to predict the 100 new sample points. The evaluation of the model’s fitting performance during the training process involves the use of the Mean Squared Error (*MSE*) and the coefficient of determination (*R*^*2*^). The equations for *MSE* and *R*^*2*^ are presented below:

**Fig 26:**
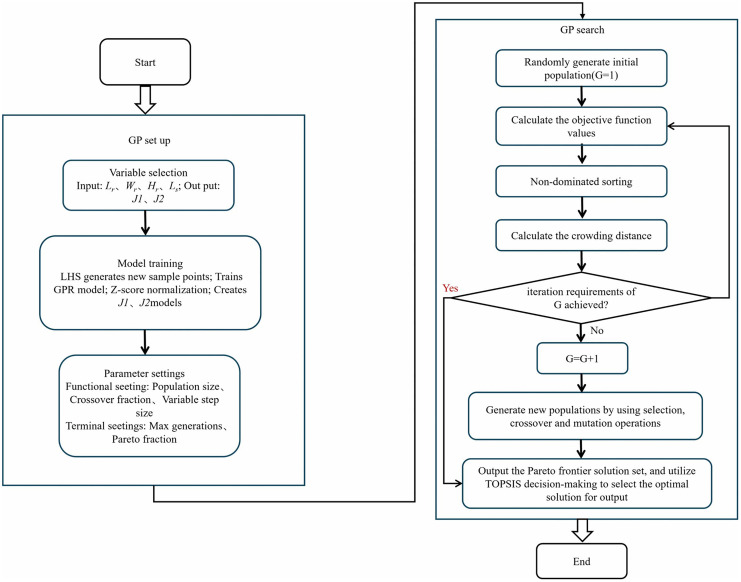
Flowchart of the NSGA-II implementation.


$$MSE=\frac{\text{1}}{N}{\overset{N}{\underset{i-1}{\sum }}\left(y_{i}-y_{i,ANN}\right)}^{\text{2}}$$ (26)

$$R^{\text{2}}=\sqrt{\frac{\overset{N}{\underset{i-1}{\sum }}{\left(y_{i,ANN}-\overset{―}{y}\right)}^{\text{2}}}{\overset{N}{\underset{i-1}{\sum }}{\left(y_{i}-\overset{―}{y}\right)}^{\text{2}}}}$$ (27)

A smaller *MSE* and an *R*^*2*^ value close to 1 indicate superior performance of the prediction model. Z-score standardization is employed to enhance both the performance and stability of the algorithm. Additionally, the Levenberg-Marquardt [70] algorithm is utilized for training. In this study, simulation experiments were conducted using MATLAB 2024b, and the results of the model training are illustrated in ():

**Fig 27:**
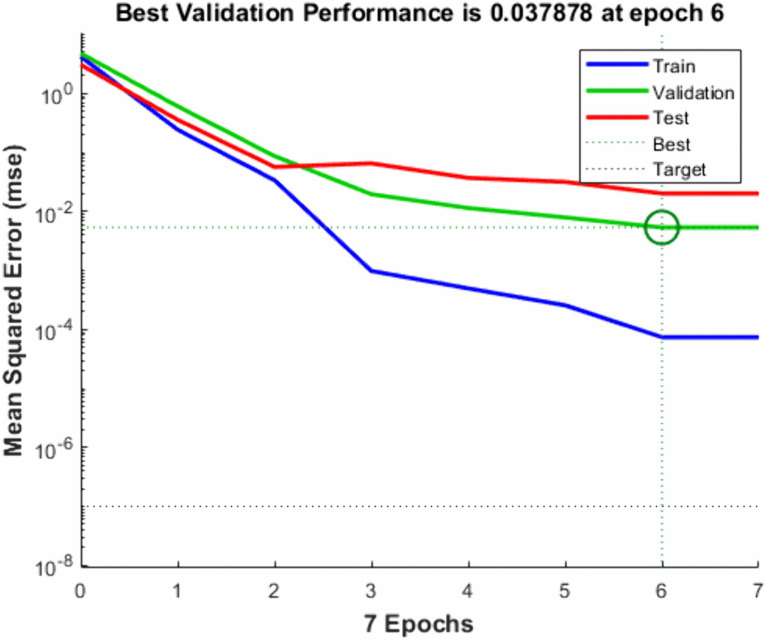
Neural network training error *MSE* result.

**Fig 28:**
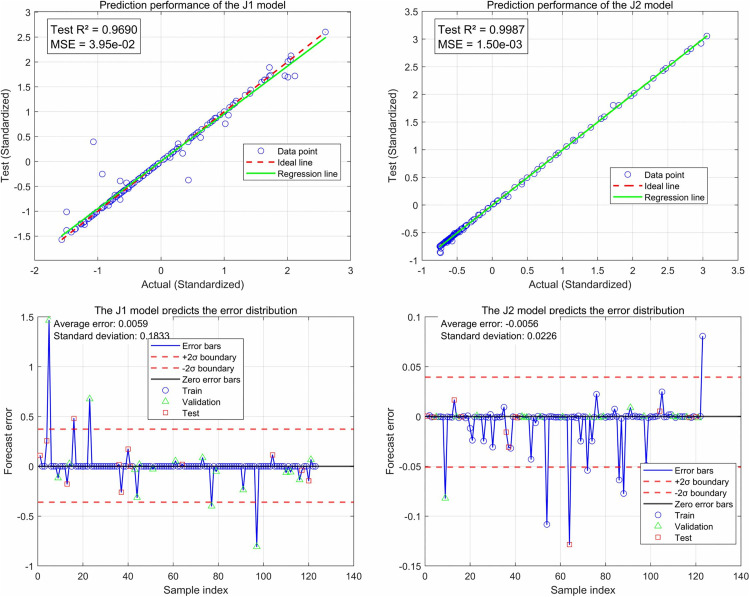
Neural network prediction results.

**Fig 29:**
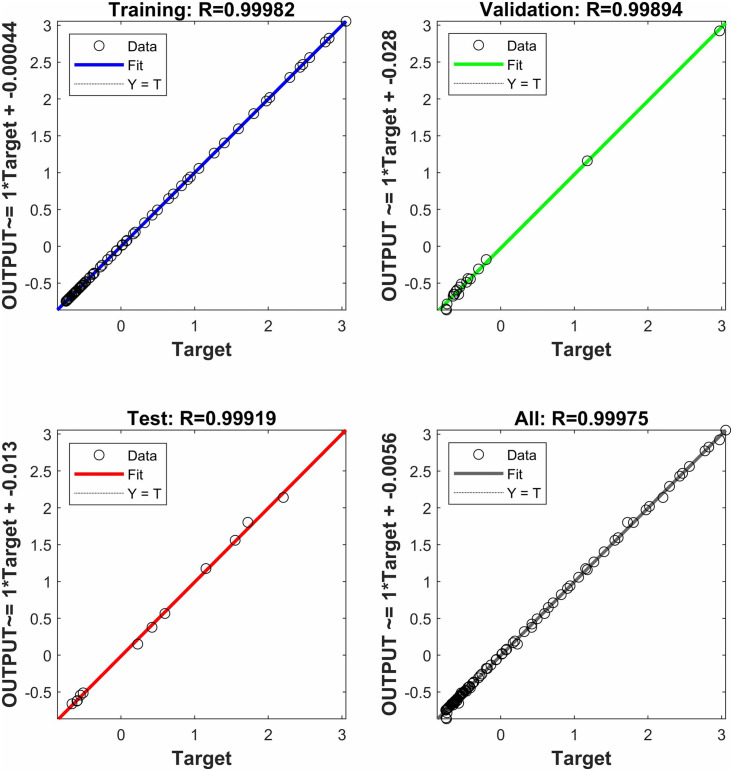
Neural network model fitting results.

To verify the accuracy of the training results, seven sets of parameter combinations were randomly designed within the specified parameter range. The corresponding test results, denoted as *PEC*, were calculated using CFD software. Subsequently, these test results were compared with the training results, referred to as *PEC*_*Te*_, as illustrated in Table 6:

**Table 6:** Randomized data training results and test results.

	1	2	3	4	5	6	7
*L*_*r*_(mm)	1.6	2.3	2.1	1	1.9	2.4	1
*W*_*r*_(mm)	1	0.6	1.3	1	1.2	0.8	0.7
*H*_*r*_(mm)	2.5	3.1	2.2	2.3	2.7	3.3	2.6
*L*_*s*_(mm)	4.5	1.9	5.9	2.1	4.4	5.7	5
*J1*	1.26	1.22	1.34	1.23	1.4	1.3	1.16
*J1*_*Te*_	1.28	1.28	1.29	1.21	1.33	1.23	1.2
*e*_*1*_	1.6%	5%	3.9%	1.7%	5.3%	5.7%	3.4%
*J2*	1.96	1.93	1.79	1.8	1.8	3.01	1.68
*J2*_*Te*_	2.01	1.87	1.7	1.84	1.72	2.86	1.77
*e*_*2*_	2.6%	3.2%	5.3%	2.2%	4.7%	5.2%	5.4%

**Fig 30:**
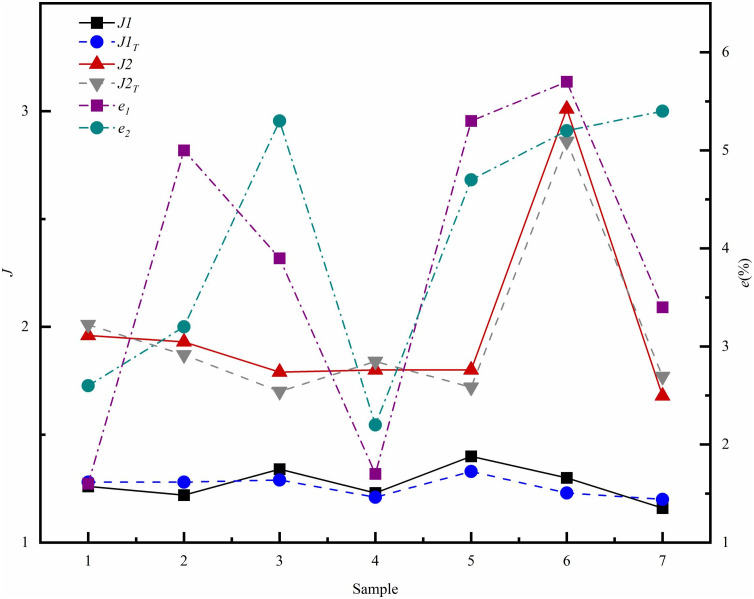
Training results (*J1*_*Te*_, *J2*_*Te*_) and test results (*J1*, *J2*), together with corresponding error curves (*e*_*1*_, *e*_*2*_).

Based on the random training results and the seven test results presented in Table 6, (Fig 30) illustrates the relationship curves between *J1*, *J2*, and the corresponding errors. The figure shows some discrepancy between the training outcomes and the test calculation results, with a maximum error of 5.7%. This level of error is considered acceptable for the surrogate model in this optimization context. This finding suggests that the model adequately represents the nonlinear problem to a certain extent.

**Fig 31:**
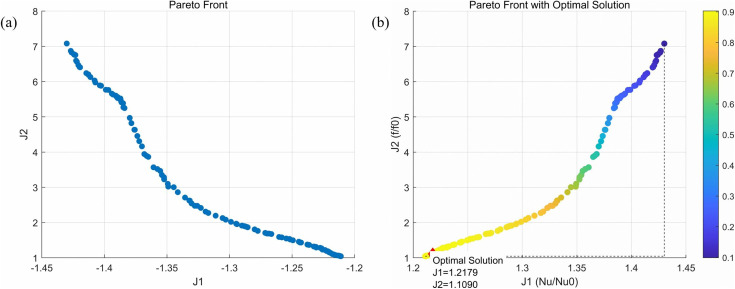
Pareto Frontier Comparison Chart. **(a)** The obtained Pareto front, **(b)** Optimal compromise solution using TOPSIS.

In this study, the NSGA-II [71]algorithm was employed for multi-objective optimization. This algorithm iteratively searches for the optimal solution, with the parameter settings detailed in Table 7. By integrating the trained ANN proxy model, the resulting Pareto front is illustrated in (Fig 31a). As one progresses to the right along the Pareto front, a gradual decline in heat transfer performance is observed, while hydraulic performance exhibits a steady improvement. To achieve an optimal balance between hydraulic and thermal performance, the TOPSIS decision-making technique is utilized to identify a compromise solution. The findings presented in (Fig 31b) indicate that the parameters Lr, Wr, Hr, and Ls are measured at 2.4928 mm, 1.3944 mm, 1.3490 mm, and 2.9742 mm, respectively, corresponding to performance metrics J1 (1.2179), J2 (1.1090), and PEC (1.18). To validate the optimized results, numerical simulations were conducted using the micro-channel featuring the identified optimal parameter set at Re = 11353. The numerical analysis reveals that the three key performance indicators of the optimized micro-channel, J1, J2, and PEC, are 1.202, 1.1535, and 1.156, respectively. The relative discrepancies for these metrics are calculated at 1.32%, 4.01%, and 2.08%, all of which fall below the 5% threshold, thus confirming the reliability of the optimization outcomes.

**Table 7:** Parameter settings for NSGA-II.

Parameter	Value
Population size	300
Max generations	150
Crossover fraction	0.85
Pareto fraction	0.4
Variable step size	0.1

### 4.3. Comparison between the optimal micro-channel design and the initial micro-channel

**Fig 32:**
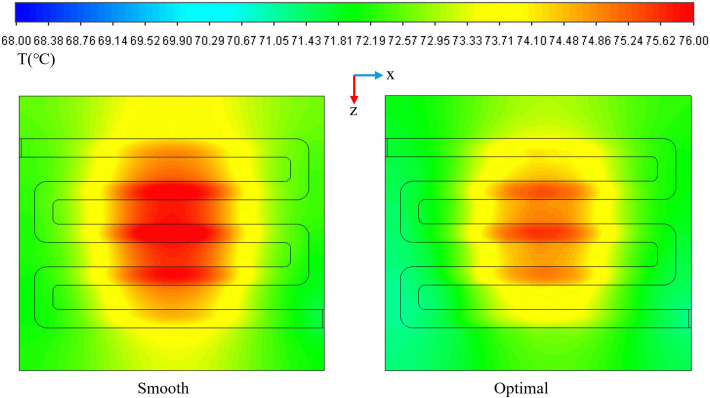
Temperature distribution map of the bottom surface of the flow channel.

**Fig 33:**
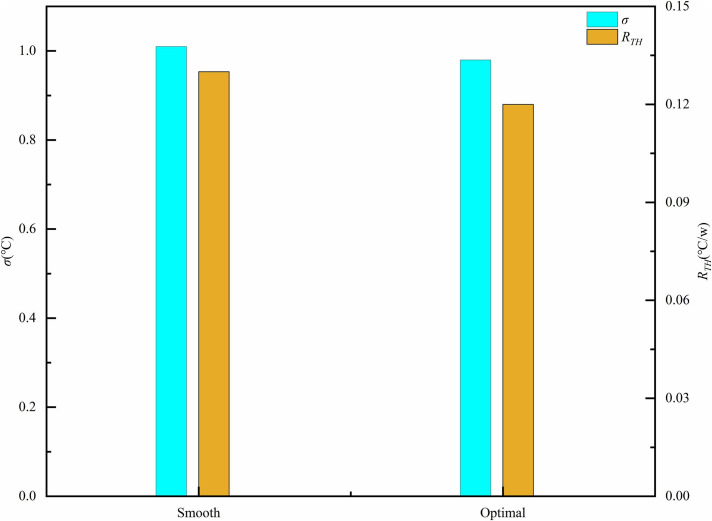
A comparison between the smooth and optimal micro-channels based on standard deviation and thermal.

**Fig 34:**
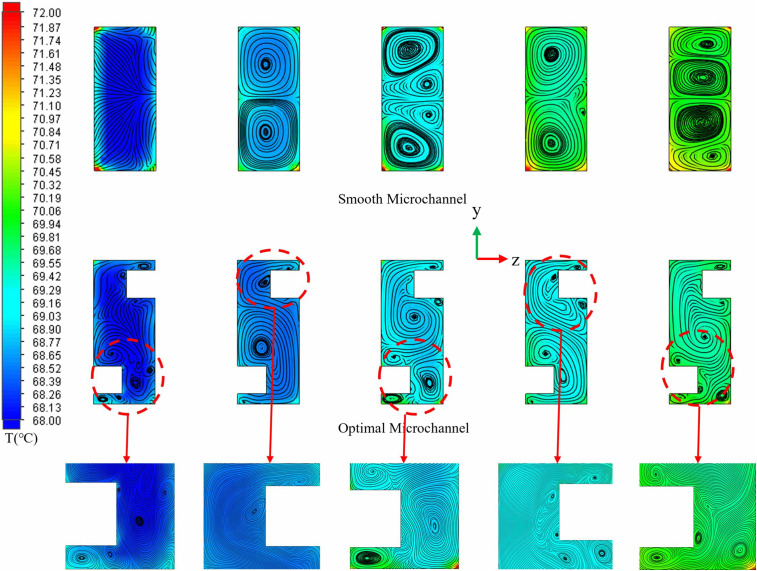
Comparison of the temperature and streamlines contours at the cross sections of *x* = 21 mm at *Re* = 11353.

**Fig 35:**
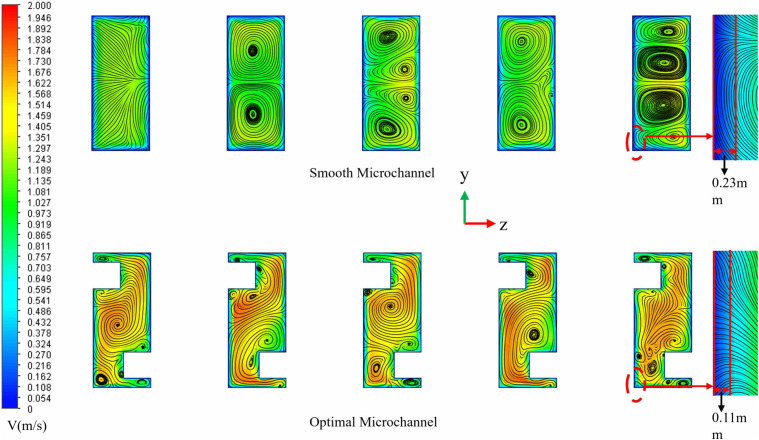
Comparison of the velocity and streamlines contours at the cross sections of *x* = 21 mm at *Re* = 11353.

**Fig 36:**
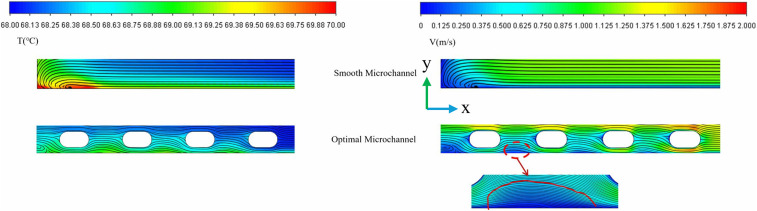
Comparison of the velocity and streamlines contours at the segmented longitudinal sections of *z* = 7.7 mm at *Re* = 11353.

(Fig 32) presents the temperature contour map of the micro-channel bottom surface derived from simulation data. (Fig 33) provides a comparative analysis of thermal resistance and temperature uniformity between smooth and optimal micro-channels. As illustrated in (Fig 32), the temperature gradient in the central region of the smooth channel is notably higher than that of the optimal structure channel. This gradient serves as an indicator of temperature uniformity within the system; it is evident that the temperature uniformity on the right is superior to that on the left. This improvement is attributed to the increased heat transfer area, which facilitates heat conduction through the internal structure of the fins to a larger surface area for dissipation. Furthermore, in the smooth channel, the flow boundary layer and thermal boundary layer continue to grow and stabilize within the region, necessitating that heat traverse this relatively thick and stable thermal boundary layer before being transferred to the main fluid flow, resulting in elevated thermal resistance. Conversely, the fins compel the main fluid flow to change direction, accelerate, and decelerate, periodically disrupting the flow and the stable development of the thermal boundary layer. This action reduces the boundary layer thickness and, consequently, thermal resistance. The boundary layer continuously redevelops at the leading edge or top of the fins, enhancing its integration with the fluid for efficient heat dissipation.

(Figs 34-36) illustrates the temperature, velocity, and streamline distribution contour plots for both the smooth channel and the optimized channel at the cross-section located at *x* = 21 mm and *z* = 7.7 of *Re* = 11353. As shown in (Figs 34 and 35), the smooth channel displays stable, spatially symmetric vortex pairs, with hot fluid near the wall and cold fluid at the center of the channel contained within their respective vortices. The efficiency of radial mixing is low due to the large and stable vortices, which hinder effective wall scraping and disrupt the thermal boundary layer adjacent to the wall. As shown in (Fig 36), heat transfer predominantly occurs through slow molecular diffusion across the thick thermal boundary layer. The presence of this thick boundary layer in the smooth channel results in reduced heat transfer efficiency. However, the introduction of capsule-shaped fins disrupts the original symmetry of the flow field. In the regions adjacent to the fins, numerous small-scale vortices are generated, which are significantly smaller than the dimensions of the channel. These vortices penetrate and disturb the boundary layer directly, thereby decreasing the primary thermal resistance to heat transfer and enhancing heat transfer efficiency. As shown in Table 8, compared to the smooth micro-channel, the optimized micro-channel exhibits a 3.1% improvement in temperature uniformity (*σ*), an 8.3% reduction in thermal resistance (*R*_*TH*_), and a 20.2% increase in the *Nu*. Although the *f* increased by 15.3%, the *PEC* improved by 15.6%. These results indicate that the optimized micro-channel exhibits higher heat transfer efficiency and achieves superior overall performance.

**Table 8:** Comparison of *σ*, *R*_*TH*_, *Nu*, *f*, *e* between smooth and optimal micro-channel.

	*σ* (°C)	*R*_*TH*_ (°C/w)	*Nu*	*f*	*PEC*
Smooth	1.01	0.13	99	0.000997	–
Optimal	0.98	0.12	119	0.00115	1.156
*E*	3.1%	8.3%	20.2%	15.3%	–

## 5. Conclusion

This study presents a systematic numerical analysis and multi-objective optimization of a novel microchannel heat sink (MCHS) featuring obliquely arranged capsule-shaped fin structures. By integrating computational fluid dynamics (CFD) with a hybrid machine learning framework, which integrates an artificial neural network (ANN) based on Gaussian process regression (GPR) with a non-dominated sorting genetic algorithm II (NSGA-II), the geometric parameters (*L*_*r*_, *W*_*r*_, *H*_*r*_, *L*_*s*_) were optimized to enhance thermal-hydraulic performance under turbulent flow conditions (*Re* range: 11353–17353). Key findings and contributions are summarized as follows:

1. The proposed capsule-shaped rib design demonstrates a superior synergistic effect on flow modulation and heat transfer. The streamlined leading edge effectively minimizes flow stagnation zones compared to traditional geometries. Furthermore, the diagonal arrangement induces distinct longitudinal vortices and enhances the local jet impingement effect. These flow structures significantly disrupt the thermal boundary layer and promote vigorous mixing between the near-wall hot fluid and the core cold fluid, thereby augmenting the convective heat transfer coefficient.
2. Both the *Nu* and *f* increase with increases in *W*_*r*_ and *H*_*r*_, but initially rise and then decline with increasing *L*_*r*_ and *L*_*s*_. The *PEC* also shows a trend of first increasing and then decreasing with all four structural parameters of the fins, which reveals the mechanism of competition between eddy currents and flow resistance.
3. By integrating neural networks and genetic algorithms, the Pareto front was obtained at *Re* = 11,353. Considering both thermal and hydraulic performance, the optimal solution was identified as *L*_*r*_ = 2.4928 mm, *W*_*r*_ = 1.3944 mm, *H*_*r*_ = 1.3490 mm, and *L*_*s*_ = 2.9742 mm. The micro-channel demonstrates excellent overall performance. In summary, this paper proposes a ribbed micro-channel design method.
4. Compared to a smooth micro-channel, the optimized micro-channel demonstrates significant performance enhancements: the *Nu* increased by 20.2%, and thermal *R*_*TH*_ decreased by 8.3%, the *σ* was reduced by 3.1%, despite the *f* increased by 15.3%, the *PEC* improved by 15.6%. These results indicate that the optimized micro-channel exhibits higher heat transfer efficiency and achieves superior overall performance.

This study is confined to the examination of single-phase water cooling using deionized water as the coolant, without exploring advanced cooling media such as nanofluids. The optimization of the fin structure is performed at a *Re* of 11353 and does not include a wider spectrum of operating conditions. Furthermore, practical manufacturing processes and production costs are not addressed. The research is limited to steady-state conditions, omitting an analysis of transient thermal responses, and does not assess the effects of long-term operation on micro-channel performance arising from scaling and corrosion. Nonetheless, the qualitative theoretical results obtained and the proposed research directions provide valuable insights for related studies.

This study primarily focuses on verifying the effectiveness of the new structure and elucidating its physical mechanisms. Future research will aim to expand the dimensionality of the optimization problem and investigate the application of optimization techniques, such as genetic algorithms, for the simultaneous and collaborative optimization of rib shape and spatial arrangement. This will entail the development of more flexible parametric geometric models and more efficient optimization strategies, with the objective of discovering microchannel configurations that exhibit superior performance across a broader design space, thereby further advancing the design of enhanced heat transfer structures toward greater intelligence and automation.

### Nomenclature

**Table:** 

*A*	Area (m^2^)
*A*_*1*_	Cross-sectional area perpendicular to heat transfer (m^2^)
*A*_*2*_	Effective convective heat transfer area of the wall (m^2^)
*A*_*m*_	Total area of the wetted surface (m^2^)
*b*	Channel depth (m)
*C*_*p*_	Specific heat J(kg·K)
*c*	Channel width (m)
*D* *D*_*1*_	Hydraulic diameter (m) Feature length (m)
*e*	Relative error
*F*_*x,*_ *F*_*y*_*, F*_*z*_	Body force acting on the fluid element (N)
*f*	Friction factor
*H*	Height (mm)
*H*_*r*_	Rib height (mm)
*h*	Convective heat transfer coefficient (W/(m^2^·K)
*J*_*1*_ *, J*_*2*_	Objective functions
*K_n_*	Knudsen Number
*k*	Coefficient of thermal conductivity
*L*	Length (mm)
*L*_*r*_	Rib length (mm)
*l*	channel cross-sectional area (m)
*M*	Random parameters/Grid numbers
*Nu*	Nusselt number
*P*	Pressure (kPa)
*PEC*	Performance evaluation coefficient
*Pr*	Prandtl number
*Q*	Heat transfer (W)
*Q*_*1*_	Total heat transferred between media (W)
*Q*_*2*_	Convective heat transfer (W)
*R* ^*2*^	Coefficient of determination
*Re*	Reynolds number
*R*_*TH*_	Thermal resistance (K/W)
*S*_*T*_	Volumetric heat source and viscous dissipation
*T*	Temperature (K)
*t*	Time (s)
*tf*	Temperature of fluid (°C)
*tw*	Temperature of wall (°C)
*u, v, w*	Velocity vectors in x, y, z directions (m/s)
*V*	Velocity (m/s)
*V*_*in*_	Inlet velocity (m/s)
*W*	Width (mm)
*W*_*r*_	Rib width (mm)
*X*	Temperature value on each grid surface (°C)
*X*_*0*_	Average value of each grid surface (°C)
*x, y, z*	Cartesian coordinates
Greek letters	
*γ*	Average free path of fluid molecules (m)
*ΔP*	Pressure drop (kPa)
*δ*_*T*_	Average absolute temperature difference (°C)
*λ*	thermal conductivity (W/(m·k))
*μ*	Dynamic viscosity (Pa·s)
*ρ*	Density (kg/m^3^)
*σ*	Temperature standard deviation (°C)
*σ*_*t*_ */σ*_*x*_	Temperature gradient (K/m)
*τ*	Viscous stress (Pa)
*τ*_*xx*_ *, τ*_*xy*_ *, τ*_*yx*_ *, τ*_*yy*_	viscous stress of the fluid element (Pa)
*τ*_*xz*_, *τ*_*zx*_*, τ*_*zy*_*, τ*_*yz*_	viscous stress of the fluid element (Pa)
*τ*_*zz*_	viscous stress of the fluid element (Pa)
Subscripts	
*0*	Reference/smooth channel
*f*	Fluid
*in*	Inlet
*max*	Maximum
*min*	Minimum
*out*	Outlet
*r*	Rib
*s*	Spacing
*Te*	Test
*w*	Wall
*x, y, z, w, μ, ν*	*x-, y-, z-, w-, μ-, ν-direction*

## Abbreviations

ANN: Artificial Neural Network
CFD: Computational Fluid Dynamics
HTC,MCHS MSE, NSGA-II, TIM,TOPSIS: Heat Transfer Coefficient,Micro-Channel Heat Sink, Mean Squared Error, Non-dominated Sorting Genetic Algorithm II, Thermal Interface Material, Technique for Order Preference by Similarity to Ideal Solution
